# Mitochondrial genomic variation, evolutionary dynamics, and phylogeny of forest-associated *Phytophthora* species in the Qinling Mountains of China

**DOI:** 10.3897/imafungus.17.203360

**Published:** 2026-08-20

**Authors:** Yijia Sun, Xiaowei Chen, Wenxin Wang, Yaling Li, Zengqiang Qian, Xiaokui Ma, Ayaka Hieno, Koji Kageyama, Mingzhu Li

**Affiliations:** 1 The Key Laboratory of Medicinal Resources and Natural Pharmaceutical Chemistry, The Ministry of Education, Shaanxi Normal University, Xi’an 710119, China College of Life Sciences, Shaanxi Normal University Xi'an China; 2 College of Life Sciences, Shaanxi Normal University, Xi’an 710119, China The Key Laboratory of Medicinal Resources and Natural Pharmaceutical Chemistry, The Ministry of Education, Shaanxi Normal University Xi'an China; 3 Center for Environmental and Societal Sustainability, Gifu University, Gifu 501-1193, Japan Center for Environmental and Societal Sustainability, Gifu University Gifu Japan

**Keywords:** Comparative mitogenomics, divergence, phylogenomics, *
Phytophthora
*

## Abstract

The genus *Phytophthora (Oomycota)* comprises highly destructive and widely distributed plant pathogens that threaten global agriculture and forest ecosystems; however, the evolutionary significance of intraspecific and interspecific mitogenomic variation within this genus remains poorly understood. In this study, complete mitochondrial genomes of 11 *Phytophthora* species, isolated from forest tree rhizospheres in the Qinling Mountains of China, were sequenced, assembled, and annotated to resolve their genetic diversity and evolutionary patterns. All 11 mitogenomes possess typical circular topologies, ranging in size from 37,502 bp (*P. citrophthora*) to 41,542 bp (*P. cryptogea*), with a uniform GC content averaging approximately 21.96%. The gene repertoire was highly conserved across all genomes, containing 34 core protein-coding genes (PCGs), two rRNA genes, 24–25 tRNA genes, and no introns. Selective pressure analysis showed that Ka/Ks ratios for all 34 core PCGs were significantly below 1 (ranging from 0.0096 to 0.1737), demonstrating that purifying selection drives mitochondrial genome evolution in *Phytophthora*. Combined mitogenomic and multi-locus phylogenomics clarified the phylogenetic placement of these taxa and provided timescale estimates for their divergence. Overall, this work enriches the mitogenomic resources of *Phytophthora* from pristine natural ecosystems and establishes a critical genomic baseline for accurate species identification, taxonomic revisions, and the study of evolutionary dynamics in oomycetes.

## Introduction

*Phytophthora*, a genus established by Anton de Bary in 1876 with *Phytophthora infestans* as the type species (De Bary 1876), belongs to the order *Peronosporales* within the *Oomycetes* and comprises some of the most destructive plant pathogens globally. Species within this genus are widely distributed across diverse ecosystems, infecting forest trees, agricultural crops, vegetables, and ornamental plants. As important soilborne pathogens and invasive organisms, they not only cause severe socio-economic losses in agriculture but also drive widespread dieback and decline in natural forest ecosystems (Lamour 2013; Jung et al. 2018; Brasier et al. 2022; Chen et al. 2022). In China, systematic research on *Phytophthora* began in the mid-20^th^ century. Ho (1996) listed 26 known species from China, including three newly described taxa. Over the following decades, extensive regional surveys in wild and agricultural environments across China significantly expanded this baseline, including the surveillance of rubber plantations on Hainan Island (Zeng et al. 2008), forest ecosystems in Yunnan Province (Huai et al. 2013), and wild apple forests in Xinjiang (Xu et al. 2019). More recently, surveys of water systems in Beijing discovered several newly recorded species, including *P. gonapodyides*, *P. borealis*, and *P. parsiana* (Cao et al. 2017). To date, a total of 41 *Phytophthora* species have been recorded in the *Catalogue of Life China* (2024 Edition), and the number of known species continues to rise rapidly, reflecting an oomycete community in China that is highly diverse yet still underestimated.

Rosenbaum (1917) first proposed dichotomous keys based on morphology—using features such as the size and shape of sporangia, the size and number of oogonia, types of antheridia, and the presence and size of chlamydospores to distinguish species. However, morphological identification of *Phytophthora* often proves challenging due to overlapping characters and structural convergence among closely related taxa. Consequently, contemporary taxonomy relies heavily on integrating morphological taxonomy with multi-locus molecular phylogenetics, a paradigm pioneered by Jung et al. (1999) and comprehensively refined by Jung et al. (2024). The implementation of multi-locus molecular markers has substantially advanced phylogenetic reconstruction within the genus. Martin et al. (2014) combined seven nuclear and four mitochondrial genes to analyze 90 formally designated taxa, resolving the genus into 11 major clades. Yang et al. (2017) expanded this framework by incorporating 142 formally described and 43 informal species. Recent taxonomic revisions have further refined these lineages, such as partitioning Clade 9 into four subclades, with *P. docyniae*, *P. macrochlamydospora*, *P. quininea*, and *P. richardiae* assigned to Subclade 9c. Phylogenetic analyses have enabled the description of over 200 species, resolving previously hidden evolutionary relationships (Chen et al. 2022; Jung et al. 2022; Abad et al. 2023). Most recently, a landmark monograph by Jung et al. (2024) described 43 new taxa, bringing the total number of formally described species within the genus to 262. Key genetic markers routinely utilized for species discrimination include the internal transcribed spacer region (ITS-rDNA), 28S ribosomal DNA (28S-rDNA), 60S ribosomal protein L10, cytochrome c oxidase subunits 1 and 2 (*cox1*, *cox2*), translation elongation factor 1-alpha (*tef1-α*), beta-tubulin (*β-tub*), enolase, heat shock protein 90 (*hsp90*), and the *tigA* gene fusion protein (Riethmüller et al. 2002; Martin and Tooley 2003; Lévesque and de Cock 2004; Villa et al. 2006; Blair et al. 2008; Robideau et al. 2011; Martin et al. 2014; Yang et al. 2017; Abad et al. 2023). Yang and Hong (2018) demonstrated the genus-wide utility of several loci across 171 *Phytophthora* species, recommending *cox1*, ITS-rDNA, and *tigA* specifically for Clade 9. Additionally, the ras-related protein (*ypt1*) gene, which exhibits high interspecific polymorphism, has successfully driven the development of species-specific primers (Schena and Cooke 2006; Schena et al. 2008; Li et al. 2011; Bi et al. 2019).

Despite these molecular advances, boundaries within species complexes remain unresolved. A prominent example is the *P. cactorum* complex, which encompasses tightly clustered taxa such as *P. hedraiandra*, *P. pseudotsugae*, and the hybrid *P. × serendipita*; their extreme morphological and genetic similarities hamper reliable diagnosis (Bourret et al. 2022; Lin et al. 2025). The rapid advancement of next-generation sequencing (NGS) has yielded a wealth of complete mitochondrial genomes, offering an exhaustive source of genetic information that significantly refines our understanding of oomycete evolution (Grayburn et al. 2004; Avila-Adame et al. 2006; Martin et al. 2007; O’Brien et al. 2014; Tangphatsornruang et al. 2016). Characterized by maternal inheritance, high copy number, low recombination rates, conserved gene content, and rapid sequence evolution, the mitogenome serves as an ideal tool for phylogenetic reconstruction, divergence time estimation, and structural rearrangement analysis (Aguileta et al. 2014; Sandor et al. 2018). Their compact structure compared with nuclear genomes also makes sequencing and comparative analysis easier. Within this organellar framework, individual mitochondrial markers—such as *cox1*, *cox2*, *nad9*, *rps10*, nad1 and *secY*—excel in resolving fine-scale evolutionary relationships, with *cox1* gaining the widest adoption due to its robust amplification efficiency and high resolution (Robideau et al. 2011; Choi et al. 2015; Jung et al. 2022, 2024). Importantly, mitochondrial genomes also provide strong phylogenetic information for solving difficult species complexes. Comparative analysis of the *P. cactorum* complex showed that despite high conservation in mitogenome size, nucleotide composition, gene order, and codon usage, phylogenomic pipelines successfully resolved interspecific boundaries, highlighting genes like *nad9* as highly promising diagnostic markers (Lin et al. 2025).

Driven by high-throughput sequencing, comparative mitogenomics of oomycetes has accelerated. Complete mitogenomes are now available for species from multiple key genera, including *Phytophthora*, *Pythium*, *Plasmopara*, *Bremia*, *Saprolegnia*, and *Achlya*, shedding light on organellar evolution across these diverse microbial lineages (O’Brien et al. 2014; Tangphatsornruang et al. 2016; Winkworth et al. 2022). These studies indicate that while oomycete mitogenomes share a conserved core of protein-coding genes, substantial variation exists among lineages regarding genome size, repeat content, intergenic length, tRNA repertoires, and synteny blocks (Martin et al. 2007; O’Brien et al. 2014). To date, 44 complete *Phytophthora* mitogenomes have been assembled, spanning 11 major clades (Winkworth et al. 2022). These genomes typically exist as circular molecules ranging tightly from 37.9 to 39.9 kb with a highly conserved core suite of respiratory-chain, ribosomal protein, and RNA genes, reflecting rigid evolutionary constraints. Although gene order remains largely syntenic across major clades, recent studies have found lineage-specific structural dynamics, including local inversions, translocations, expanded intergenic regions, and divergent repeat landscapes, proving that mitogenome evolution within the genus is not entirely static (Yuan et al. 2017; Cai and Scofield 2020; Lin et al. 2025).

Current studies show that many devastating agricultural and forestry epidemics driven by *Phytophthora* lineages originated in natural ecosystems (Jung et al. 2017, 2020, 2024). Evidently, natural forests serve as evolutionary reservoirs for pathogen diversification, host adaptation, and the generation of novel lineages. Surveys in rhizosphere soils of undisturbed forests and riparian ecosystems continue to unearth previously unknown or cryptic *Phytophthora* species, underscoring the vast uncharacterized diversity hidden in natural habitats (Jung et al. 2017, 2020, 2022, 2024; Horta Jung et al. 2025; Bregant et al. 2025). In China, however, severe economic losses caused by *Phytophthora* diseases in crop production have heavily skewed research toward agricultural epidemiology, functional pathogenesis, host-pathogen interactions, and agronomic management (Li et al. 2017; Chen et al. 2023; Liu et al. 2025). While extensive surveys in natural ecosystems globally have advanced our understanding of the morphological and physiological adaptations of forest-associated *Phytophthora* species (Jung et al. 2011, 2017, 2022, 2024; Scanu et al. 2015; Burgess et al. 2018; Dangi et al. 2021), such investigations remain highly limited in critical ecological barriers and biodiversity hotspots like the Qinling Mountains. Furthermore, the comparative mitogenomics and molecular mechanisms underlying their long-term genetic differentiation remain poorly explored.

In fact, although several targeted *Phytophthora* surveys have been conducted in certain Chinese natural forests, such as in Yunnan Province (Huai et al. 2013) and the wild apple forests of Xinjiang Province (Xu et al. 2019), systematic diversity surveys across China’s major forest ecosystems remain limited. This is particularly true in ecologically vital mountainous zones defined by complex floristic composition and high environmental heterogeneity (Mi et al. 2021). This knowledge gap severely restricts our comprehension of the evolutionary history, biogeography, and adaptive trajectories of forest-associated lineages, hampering our capacity to forecast emerging biosecurity risks and ecological threats. As a transitional zone between northern and southern climates in China, the Qinling Mountains constitute an essential forest ecological zone. The region’s rugged topography, diverse vegetation profiles, and rich host plant resources make it a vital natural laboratory for investigating the diversity and evolution of plant-associated microbes (Lv et al. 2023; Lyu et al. 2024). Accumulating evidence suggests that *Phytophthora* populations in wild forest ecosystems maintain high genetic diversity and preserve ancestral or unique evolutionary lineages (Jung et al. 2017, 2020; Horta Jung et al. 2025). This is notably exemplified by the discovery of eight unique evolutionary lineages of *Phytophthora ramorum* in the natural forests of Northern Vietnam and Japan (Jung et al. 2021). Consequently, conducting surveys of *Phytophthora* in national natural forest reserves that encompass both northern and southern Chinese climates and vegetation types will not only enhance our understanding of oomycete diversity in China’s natural habitats but also be of paramount scientific significance for understanding the speciation and evolutionary characteristics of oomycetes.

In this study, focusing on 11 *Phytophthora* species isolated from the rhizosphere of forest trees in the Qinling Mountains, we successfully sequenced, assembled, and annotated their complete mitochondrial genomes. By integrating comparative mitogenomics, phylogenomics, and molecular clock analyses, we seek to clarify the organellar genetic diversity, divergence patterns, and evolutionary characteristics of forest-associated *Phytophthora* species. Specifically, this study is designed to: (1) comprehensively characterize the genomic content, architectural organization, and evolutionary constraints of these eleven newly sequenced mitogenomes; (2) systematically investigate regions of sequence conservation, hot spots of variability, and structural synteny across multiple *Phytophthora* clades, with a particular focus on defining novel genomic rearrangement modes; and (3) establish a robust, multilocus taxonomic framework and estimate lineage divergence times using combined mitochondrial and nuclear datasets under a calibrated molecular clock. Notably, by integrating comparative synteny with phylogenetic analyses, this work systematically classifies the mitogenomes into three distinct arrangement types that exhibit a strong, clade-specific phylogenetic signal. This discovery significantly advances our understanding of *Phytophthora* mitogenomic evolution, demonstrating that structural rearrangements are tightly linked to lineage divergence and can serve as stable macro-evolutionary markers. Consequently, this study not only enriches the available organelle genomic resources for wild oomycetes in natural forest reservoirs, but also provides a powerful comparative framework that supports future research in evolutionary biology, disease surveillance, and molecular diagnostics of forest pathogens.

## Materials and methods

### Strain isolation, DNA extraction and sequencing

Based on the leaf-baiting method (Jung et al. 1996, 2020), a total of 143 *Phytophthora* strains were isolated from rhizosphere soils of the dominant tree species in the Qinling Mountains of China (106.63°E–118.34°E, 33.11°N–35.07°N). At each sampling site, a 20 m × 20 m plot was established, within which 10 individual trees of dominant species were randomly selected for rhizosphere soil collection. All 143 isolates were molecularly identified and found to belong exclusively to 11 distinct *Phytophthora* species. Detailed information regarding geographic sampling locations, coordinates, sampled host tree species, and isolated *Phytophthora* strains for each site is summarized in Suppl. material 1: table S1. For each soil sample, approximately 200–300 g of sieved soil was placed into a sterile container and mixed thoroughly with 500 mL of sterile water. Following the sedimentation of coarse soil particles, floating debris was removed, and asymptomatic, healthy host leaves were placed on the water surface as baits. The bait leaves comprised *Metasequoia glyptostroboides*, *Parthenocissus tricuspidata*, *Torreya grandis*, *Lindera glauca*, *Magnolia* spp., *Cornus officinalis*, *Aesculus chinensis*, *Acer palmatum*, *Chamaecyparis obtusa*, *Platycladus orientalis*, *Diospyros kaki*, *Quercus* spp., and *Cedrus deodara*. All baiting cultures were incubated in darkness at 20 °C for 3–7 d. Upon the development of water-soaked lesions or necrotic spots, infected leaf tissues were harvested, rinsed with sterile water, and blotted dry using sterile filter paper. Small tissue fragments (~ 2 × 2 mm) excised from the margins of active lesions were transferred onto V8 selective medium. Plates were incubated at 20 °C in darkness for 2–7 d. Hyphal tips from the advancing margins of developing colonies were sequentially transferred onto fresh V8 selective medium for purification and maintenance. Finally, 11 representative strains including *Phytophthora citrophthora* 236HBY, *Phytophthora acerina* 17XS, *Phytophthora citricola* K10, *Phytophthora cactorum* 232HNP, *Phytophthora plurivora* 173MX, *Phytophthora multivora* 79TP, *Phytophthora pini* HH5, *Phytophthora himalsilva* 226ZA, *Phytophthora sansomeana* 208HY, *Phytophthora pseudocryptogea* LX10, and *Phytophthora cryptogea* 131SQ, were selected and analyzed in this study.

Total genomic DNA (gDNA) was extracted following the protocol of Kageyama et al. (2003) with modifications introduced by Li et al. (2011), incorporating a magnetic bead purification step (MagExtractor-Plant Genome; Toyobo Co., Osaka, Japan). DNA integrity was evaluated via agarose gel electrophoresis and UV spectroscopy. DNA purity and concentration were quantified using a NanoDrop One spectrophotometer (Thermo Scientific, Wilmington, USA) and a Qubit 3.0 Fluorometer (Invitrogen, Shanghai, China). To confirm the identity of the 11 *Phytophthora* species, five loci—the internal transcribed spacer region (ITS-rDNA), *cox1*, *ypt1*, *rps10*, and *tigA*—were amplified and sequenced (Blair et al. 2008), and subjected to multilocus phylogenetic analysis (Suppl. material 2: fig. S1) in combination with morphological characteristics (Erwin and Ribeiro 1996; Hansen et al. 2009; Jung and Burgess 2009; Scott et al. 2009; Hong et al. 2011; Vettraino et al. 2011; Ginetti et al. 2014; Safaiefarahani et al. 2015; Jung et al. 2024). All multi-locus nucleotide sequences were deposited in the NCBI GenBank database (accession numbers are provided in Suppl. material 1: table S2).

### Whole-genome sequencing, mitogenome assembly and annotation

High-quality gDNA samples were submitted to Novogene Co., Ltd. (Tianjin, China) for library construction and whole-genome sequencing (WGS). The gDNA was randomly fragmented using a Covaris Focused-ultrasonicator, and sequencing libraries were prepared following the standard Illumina DNA library preparation protocol. Library integrity and quality were verified using an Agilent 2100 Bioanalyzer (Agilent Technologies, CA, USA) and quantitative PCR. Validated libraries were sequenced on an Illumina NovaSeq 6000 platform, generating 150 bp paired-end reads. Raw reads were filtered to remove low-quality data according to the following criteria: paired reads were discarded if the N content exceeded 10% of the total bases, if low-quality bases (Q ≤ 5) made up more than 50% of the read, or if adapter sequences were detected.

Following adapter removal and quality trimming, high-quality reads were assembled *de novo* using GetOrganelle v1.7 (Jin et al. 2020) software to yield complete, circular mitochondrial genomes. To validate the assembly quality and coverage statistics, clean paired-end reads were mapped back to each corresponding assembled mitogenome using BWA-MEM v0.7.17 (Li 2013). Alignment statistics, coverage depth, depth uniformity, and insert-size distributions were evaluated using Qualimap v2.2.1 (Okonechnikov et al. 2016). Sequence consensus accuracy and base-call precision were further assessed using Pilon v1.24 (Walker et al. 2014) by detecting potential single nucleotide polymorphisms (SNPs) and small indels. The circular nature of each mitogenome was initially determined by GetOrganelle v1.7 and further validated by inspecting sequence alignments across the junction region. Specifically, paired-end read mapping confirmed seamless coverage continuity with spanning paired reads bridging the physical 5' and 3' terminal ends of the assembled contigs, demonstrating complete genome circularization. Detailed sequence depth, read-mapping metrics, insert-size statistics, and assembly validation results for all 11 mitogenomes are summarized in Suppl. material 1: table S3.

Protein-coding genes (PCGs), transfer RNAs (tRNAs), and ribosomal RNAs (rRNAs) were initially identified by alignment with reference *Phytophthora* mitogenomes in Geneious Prime v2025.0.2 (Auckland, New Zealand) utilizing MAFFT v7.5 under the Auto option (Katoh and Standley 2013). Open reading frames (ORFs), unidentified ORFs (uORFs), rRNAs, tRNAs, and introns across the 11 mitogenomes were predicted using MITOS (Bernt et al. 2013) based on the standard genetic code (Genetic Code 1). Secondary structures of the predicted tRNA genes were derived via MITOS using default parameters, subsequently validated with tRNAscan-SE v2.00, and rendered in Adobe Illustrator 2022. Graphical circular maps of the 11 *Phytophthora* mitogenomes were generated using the online tool OGDraw v1.2 (Greiner et al. 2019). The complete mitogenomic sequences of all 11 species have been deposited in GenBank (accession numbers are listed in Suppl. material 1: table S4).

### Sequences analyses and repetitive elements

DNA strand asymmetry across the 11 *Phytophthora* mitogenomes was quantified using the following formulas: AT skew = [A – T] / [A+T], and GC skew = [G – C] / [G + C] (Zou et al. 2022). Relative synonymous codon usage (RSCU) for all complete PCGs was calculated using custom R scripts. Codons with RSCU > 1 were defined as preferentially used. Synonymous (Ks) and nonsynonymous (Ka) substitution rates for 34 core PCGs (*atp1*, *atp6*, *atp8*, *atp9*, *cob*, *cox1*, *cox2*, *cox3*, *nad1*, *nad2*, *nad3*, *nad4*, *nad4L*, *nad5*, *nad6*, *nad7*, *nad9*, *nad11*, *rpl2*, *rpl5*, *rpl6*, *rpl14*, *rpl16*, *rps2*, *rps3*, *rps4*, *rps7*, *rps8*, *rps10*, *rps11*, *rps12*, *rps13*, *rps14*, and *rps19*) were calculated using KaKs Calculator 3.0 (Zhang 2022) based on pairwise comparisons among orthologs. Pairwise genetic distances for these 34 core PCGs were estimated in MEGA v7.0.26 based on the Kimura-2-parameter (K2P) substitution model (Caspermeyer 2016). Interspersed repeats and large intragenomic duplications within the mitogenomes were identified via BLASTN searches with an E-value of <1e-10 (Chen et al. 2015). Tandem repeats were mapped using Tandem Repeats Finder (Benson 1999). Simple sequence repeats (SSRs) were scanned using MISA (Thiel et al. 2003; Beier et al. 2017) with default minimum thresholds set to 10 for mononucleotides, 6 for dinucleotides, and 5 each for tri-, tetra-, penta-, and hexanucleotides. Mitogenomic collinearity across the 11 *Phytophthora* species was evaluated and visualized using Mauve v2.4.0 (Darling et al. 2004).

### Comparative mitogenomic analyses of *Phytophthora* species

Comparative mitogenomic analyses were performed to evaluate structural and compositional conservation and variation among the *Phytophthora* species, focusing on genome size, base composition, GC content, gene repertoires, and gene order. Variations in PCG lengths, GC content, AT skews, and GC skews were plotted and visualized using the R package ggplot2 v4.0.2 in R software v4.5.2.

### Mitogenomic phylogenetic analyses

To resolve the phylogenetic relationships and evolutionary divergence timelines of the 11 *Phytophthora* species, two independent multi-locus datasets were constructed: (1) a concatenated mitochondrial dataset encompassing 34 core PCGs from 63 *Peronosporaceae* species, and (2) a concatenated multi-locus dataset comprising five gene fragments (ITS, *cox1*, *rps10*, *ypt1*, and *tigA*) from 58 *Peronosporaceae* species. *Phytopythium vexans* and *Globisporangium ultimum* (*Pythiaceae*) were selected as outgroups. Gene sequences were aligned, trimmed, and concatenated using the integrated pipeline in PhyloSuite v1.2.3 (Zhang et al. 2020). Best-fit nucleotide substitution models and partitioning schemes were determined using ModelFinder v2.2.0 based on the Bayesian Information Criterion (BIC). Phylogenetic reconstructions were performed using both Bayesian Inference (BI) and Maximum Likelihood (ML) criteria. BI analysis for the mitochondrial dataset was executed in MrBayes v3.2.7a under the GTR + F + G4 model, running two parallel Markov chain Monte Carlo (MCMC) chains for 10,000,000 generations with sampling every 1,000 steps; the initial 25% of samples were discarded as burn-in. ML phylogenies were inferred using IQ-TREE (Nguyen et al. 2015) under the GTR + R4 + F model, with node support assessed via 10,000 ultrafast bootstrap replicates (Minh et al. 2013) and the Shimodaira–Hasegawa-like approximate likelihood-ratio test (Guindon et al. 2010).

### Molecular clock analysis and divergence time estimation

Divergence times were estimated under a Bayesian relaxed molecular clock framework implemented in MCMCtree within the PAML v4.10 package (Yang 2007), configured and executed via the MDGUI interface in PhyloSuite v2 (Zhao et al. 2025). An independent rates (IR) model was selected to model and accommodate rate variations across lineages, and site substitution models were assigned automatically per partition. The Hessian matrix was calculated using IQ-TREE under the optimized substitution models. Temporal calibration points were established based on fossil records from previous literature (Jung et al. 2022, 2024), applying uniform bounds (*B* function) to the root node and upper-bound maximum constraints (*U* function) with soft bounds to internal nodes (Suppl. material 1: table S5). Divergence dating analyses were conducted independently for both genomic datasets using identical parameter configurations. MCMC simulations were run for 50,000,000 generations, with trees sampled every 2,500 steps (sampfreq = 2500) to harvest collect a total of 20,000 samples (nsample = 20000). The initial 10% of generations were discarded as burn-in (burnin = 5, 000, 000). MCMC convergence was confirmed using Tracer v1.7.2 to verify that all primary model parameters achieved effective sample size (ESS) values > 200. Chronograms were formatted and visualized using FigTree v1.4.4. (http://tree.bio.ed.ac.uk/software/figtree/).

### Abbreviations

**Mitogenome** Mitochondrial genome

**PCG** Protein-coding gene

**rRNA** Ribosomal RNA

**tRNA** Transfer RNA

**RSCU** Relative synonymous codon usage

**SSR** Simple sequence repeat

**Ks** Synonymous substitution rates

**Ka** Nonsynonymous substitution rates

**K2P** Kimura-2-parameter

**BI** Bayesian inference

**ML** Maximum Likelihood

**ITS** internal transcribed spacer

***rps10*** ribosomal protein S10

***ypt1*** beta-tubulin; his: histone

***tigA*** translation initiation factor 1A

***rns*** small subunit ribosomal RNA gene

***rnl*** large subunit ribosomal RNA gene

## Results

### General Features of *Phytophthora* Mitogenomes

**Figure 1.**
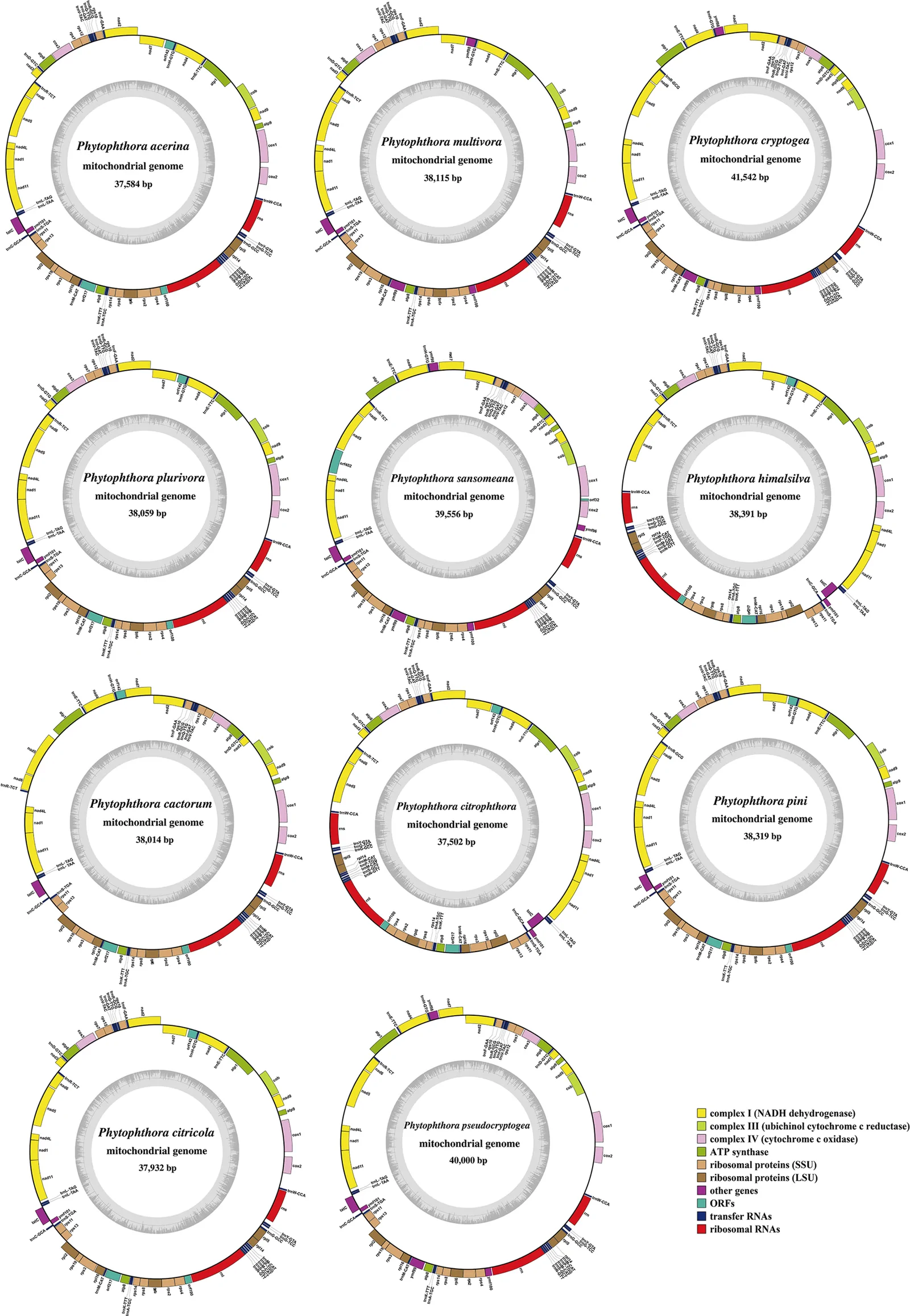
Circular maps of the eleven *Phytophthora* mitogenomes. Genes are represented by different colored blocks. Colored blocks outside each ring indicate that the genes are on the forward strand, while colored blocks within the ring indicate that the genes are located on the reverse strand. Genes on the forward strand are transcribed in a counterclockwise direction, while genes on the reverse strand are transcribed in a clockwise direction. The inner grayscale bar graph shows the GC content of the mitochondrial sequences. The circle inside the GC content graph marks the 50% threshold.

We successfully assembled and annotated the complete mitogenomes of 11 *Phytophthora* species, constructing circular genome maps to facilitate comparative analysis (Fig. 1). All 11 mitogenomes presented a typical circular architecture, with total sizes spanning from 37,502 bp (*P. citrophthora*) to 41,542 bp (*P. cryptogea*). The genomic GC content was low and uniform, ranging between 21.6% and 22.6% (avg. 22.0%). Individually, *P. pseudocryptogea* and *P. cryptogea* had the highest GC content, whereas *P. plurivora* and *P. citricola* showed the lowest (Suppl. material 1: table S6). This mitogenome size congruency aligns with their phylogenetic placements, as *P. cryptogea*/*P. pseudocryptogea* (Subclade 8a) and *P. citricola*/*P. plurivora* (Subclade 2c) represent pairs of close sister species inheriting these shared ancestral traits. Nucleotide composition analysis indicated that most species preferred positive AT and GC skews, whereas *P. himalsilva* and *P. citrophthora* stood out by displaying negative values for both skews (Suppl. material 1: table S6), reflecting their close phylogenetic relationship within Clade 2a. The detailed proportions of PCGs, introns, intergenic spaces, and non-coding RNAs (ncRNAs) are summarized in Suppl. material 1: figs S6, S7.

The mitogenomes contained 38 to 42 PCGs; *P. sansomeana* carried the highest number of PCGs, while *P. cactorum* had the fewest. Crucially, all 11 mitogenomes shared a conserved suite of 34 core PCGs (Suppl. material 1: table S7), including four ATP synthase subunits (*atp1*, *atp6*, *atp8*, and *atp9*), ten NADH dehydrogenase subunits (*nad1*–*nad7*, *nad9*, and *nad11*), five large ribosomal subunit proteins (*rpl2*, *rpl5*, *rpl6*, *rpl14*, and *rpl16*), and 11 small ribosomal subunit proteins (*rps2*–*rps4*, *rps7*–*rps8*, *rps10*–*rps14*, and *rps19*). Additionally, apocytochrome b (*cob*) and three cytochrome c oxidase subunits (*cox1*, *cox2*, and *cox3*) were universally present. Interestingly, three unique open reading frames (*orf402*, *ymf96*, and *orf32*) occurred exclusively in *P. sansomeana* and were absent in the other ten species. No introns disrupted any of these newly sequenced mitogenomes.

### RNA genes

All 11 mitogenomes contained two ribosomal RNA genes corresponding to the small (*rns*) and large (*rnl*) subunits, both of which were entirely intron-free. The length of *rnl* varied from 2,650 bp to 2,688 bp (mean 2,657 bp), with the longest found in *P. sansomeana* and the shortest in *P. cactorum* and *P. multivora*. Meanwhile, *rns* spanned from 1,501 bp to 1,506 bp (avg. 1,504 bp), reaching its maximum length in *P. multivora*, *P. plurivora*, *P. pini*, and *P. citricola* (Suppl. material 1: table S7).

**Figure 2.**
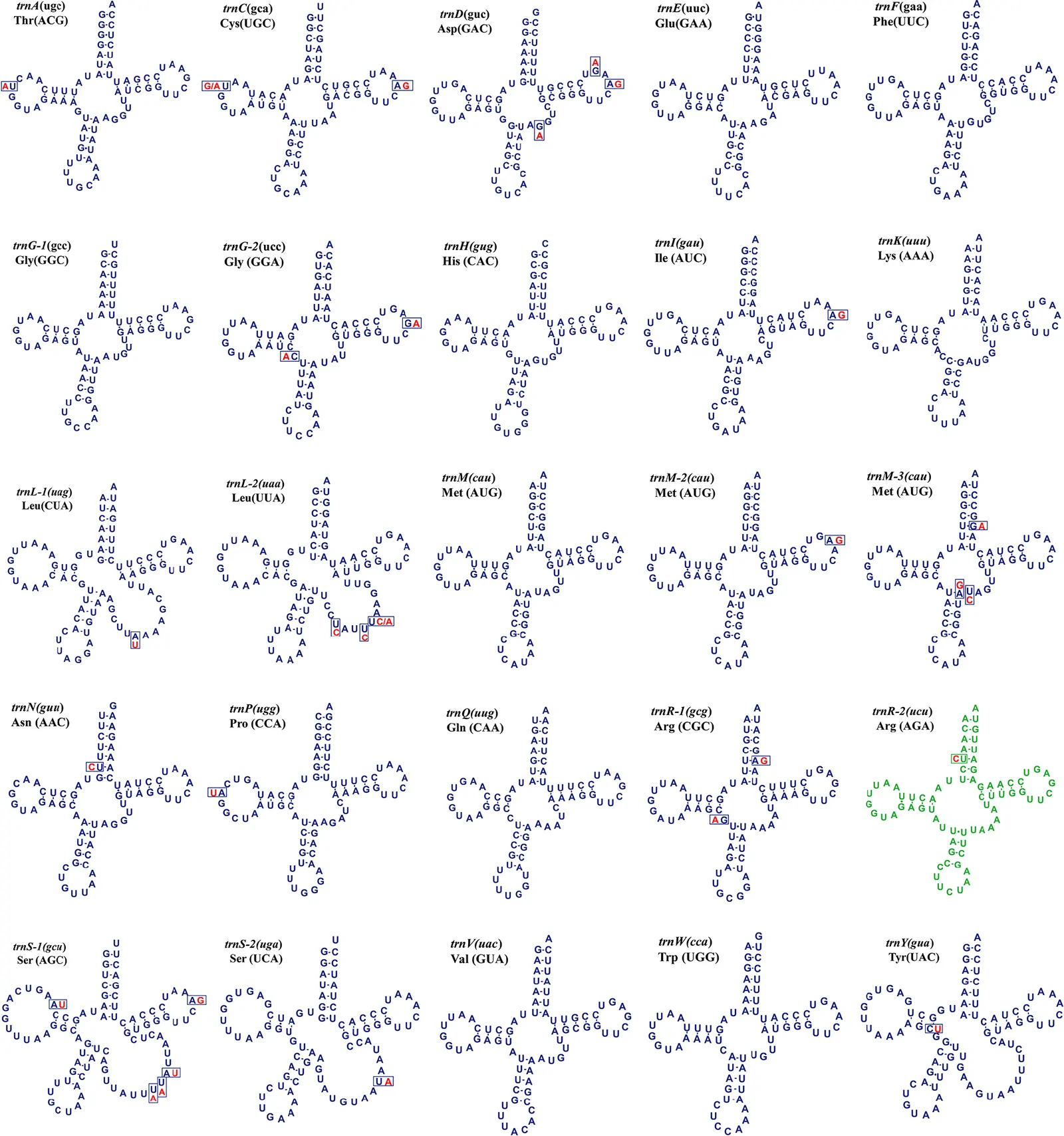
Putative secondary structures of tRNA genes identified in the mitogenomes of 11 *Phytophthora* species. The 25 tRNAs in blue font represent tRNAs shared by the 11 *Phytophthora* species, while the tRNA in green font represents a tRNA present in all species except *Phytophthora cryptogea* (strain 131SQ) and *Phytophthora pini* (strain HH5). In addition, residues conserved across the 11 mitogenomes are shown in blue, while variable sites are shown in red.

A stable complement of 24 to 25 distinct tRNA genes was identified across all species, collectively encoding 19 standard amino acids (Fig. 2). All predicted tRNAs folded into typical cloverleaf secondary structures ranging from 71 to 89 bp, with length variations stemming mostly from the variable arm (Suppl. material 1: table S7). Due to an extended variable arm, *trnS* was consistently the longest tRNA. Sixteen tRNAs featured variable positions, totaling 28 variable sites across the 11 species (Suppl. material 1: table S8). All mitogenomes contained two tRNAs for glycine, leucine, and serine (with different anticodons), alongside three tRNAs for methionine (sharing the same anticodon). Except for *P. pini* and *P. cryptogea*, all species had two tRNAs for arginine with distinct anticodons.

During tRNA secondary structure folding, we captured 32 base mismatches across all genes, comprising 30 G–U pairs and two U–U pairs distributed across different regions. G–U mismatches were common in mitochondrial tRNAs and were generally tolerated or corrected via RNA editing, while non-canonical U–U pairs likely supported RNA stability and functional diversity.

### Codon usage of PCGs

**Figure 3.**
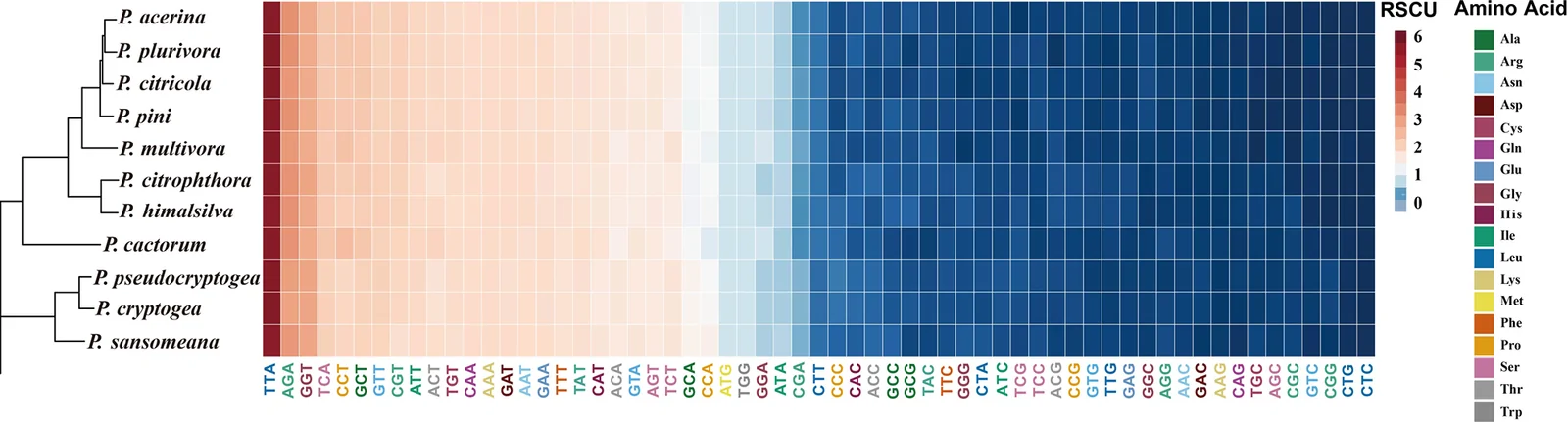
Relative synonymous codon usage (RSCU) in 11 *Phytophthora* species. The heatmap displays the RSCU values for each codon across 11 *Phytophthora* species. RSCU values are color-scaled, where warmer colors (e.g., red) indicate higher codon usage bias (RSCU > 1), and cooler colors (e.g., blue) represent lower usage bias (RSCU < 1). RSCU = 1 indicates no bias. The hierarchical clustering dendrogram on the left side reflects similarities in codon usage patterns among species, respectively.

Across all 11 mitogenomes, ATG served as the universal start codon for all PCGs, with no alternative initiation codons detected (Fig. 3). Among stop codons, TAA predominated (95.1%), followed by TGA (2.6%) and TAG (2.3%). Notably, *nad11* consistently used TGA as its stop codon in all species. In contrast, TAG usage was lineage-specific and restricted to particular genes: it terminated *rps11* and *rps13* in *P. plurivora*, *P. acerina*, and *P. citricola*; *nad9*, *rps11*, and *rps13* in *P. multivora*; and *rps13* in *P. pini*, while remaining completely absent in the other six species. Codon usage analysis highlighted a strong preference for A/T-rich codons, aligning with the low genomic GC content (21.6%–22.6%). Codons with the highest Relative Synonymous Codon Usage (RSCU) values were heavily A/T-enriched, including TTA (Leu), TTT (Phe), AAA (Lys), AAT (Asn), and ATA (Ile), all exceeding 1.9 (Suppl. material 1: table S9). Conversely, G/C-rich codons yielded exceptionally low or near-zero RSCU values. This clear bias mirrors the overall AT-rich background of the mitogenomes, indicating that codon usage bias heavily shapes nucleotide composition in *Phytophthora* .

### Repeats

We identified 324 simple sequence repeats (SSRs) across the 11 mitogenomes, consisting of 301 mononucleotide repeats (92.9%), eight dinucleotide repeats, three trinucleotide repeats, and 12 compound SSRs (Suppl. material 1: table S10). No tetra-, penta-, or hexanucleotide SSRs occurred. Mononucleotide repeats (poly-A/poly-T) predominated, with each genome containing 27 to 37 SSRs that typically spanned 10 to 15 bp. *P. cactorum* had the highest number of SSRs (37), followed by *P. citrophthora* and *P. plurivora* (31). Additionally, we detected 439 tandem repeats, ranging from 19 to 56 repeats per genome (Suppl. material 1: table S11). *P. himalsilva* showed the fewest tandem repeats, whereas *P. acerina* carried the most. Repeat units ranged from 2 to 54 bp in length, with copy numbers between 1.9 and 24.7, showing strong A/T enrichment. Dispersed repeat analysis revealed high sequence similarity (98%–99%) among species belonging to the same clade, which dropped to 91%–96% between different clades (Suppl. material 1: table S12). The distribution and composition of these repeats matched phylogenetic groupings, pointing to lineage-specific repeat evolution.

### Variations, genetic distances, and evolutionary rates of PCGs

**Figure 4.**
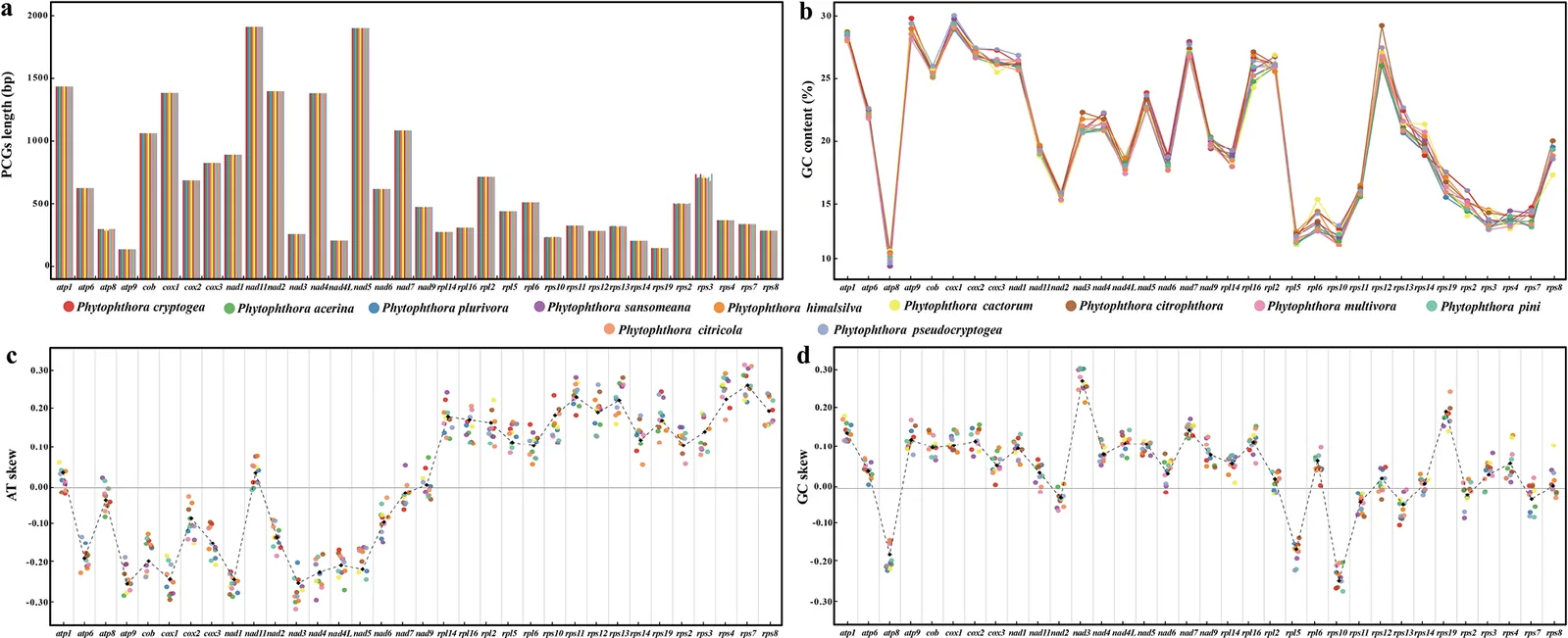
Full-length sequence variation in the length and base composition of each of the 34 protein coding genes (PCGs) among eleven *Phytophthora* mitochondrial genomes **a**PCG length variation **b** GC content across PCGs**c** AT skew **d** GC skew.

To examine sequence evolution, we assessed the gene length, GC content, and skews of the 34 core PCGs. In terms of length (Fig. 4a), *nad5* was the longest (~2,000 bp) and *cox1* followed (~1,550 bp), while *atp8* (~300 bp) and *nad4L* (~400 bp) were the shortest (Suppl. material 1: table S13). While core respiratory chain genes (*cox2*, *nad1*, *nad2*) and ribosomal protein genes (*rpl2*, *rpl5*, *rpl6*, *rpl14*) maintained fully conserved lengths, others exhibited variations. Notably, *rps3* varied the most, with lengths ranging from 774 to 831 bp (a 57-bp difference), followed by minor variations in *atp8* (381–393 bp) and *cob* (1,152–1,155 bp). The GC content of the 34 PCGs remained low (Fig. 4b), spanning from 9.4% (*atp8*) to 30.5% (*cox1*). *cox1* (avg. 29.8%) and *atp9* (29.2%) marked the highest values, whereas *atp8* (10.2%), *rps10*, and *rpl5* (11.7%) were the lowest (Suppl. material 1: table S13). Respiratory chain genes generally maintained higher GC contents than ribosomal protein genes, matching the expectation that genes under tighter purifying selection retain higher GC levels. For AT skew (Fig. 4c), respiratory chain genes displayed a consistent negative skew across all species, with *atp8*, *atp9*, *cob*, and *cox1* being the most biased. In contrast, ribosomal protein genes preferred adenine, showing a positive AT skew (*nad9*, *rpl14*, *rpl16*, *rps2*, *rps3*, and *rps4*). For GC skew (Fig. 4d), positive values emerged in almost all 34 PCGs. *cox1* showed the strongest positive GC skew (+0.14 to +0.22) across all species, while most *nad*, *rpl*, and *rps* genes also remained positive. *atp8* was the sole exception, consistently showing a highly negative GC skew, which suggests distinct mutational pressures or unique evolutionary constraints on this gene.

**Figure 5.**
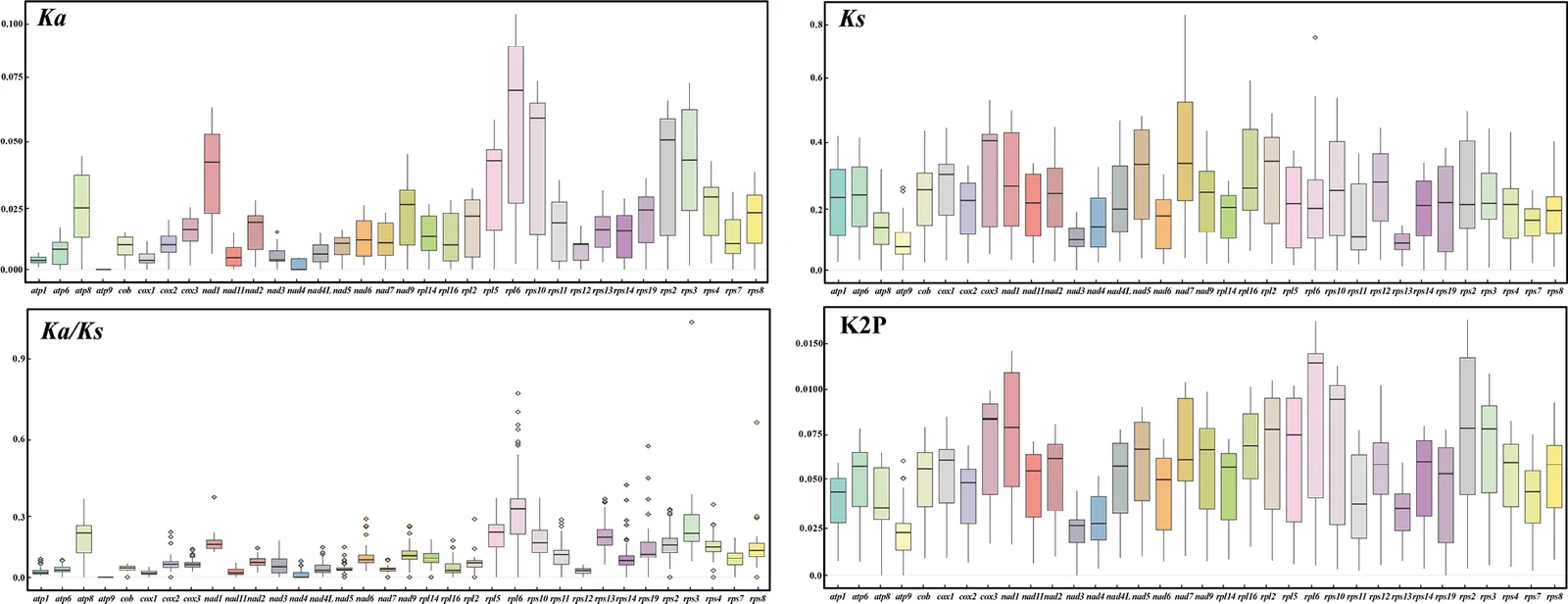
Evolutionary analysis of 34 core protein-coding genes in 11 *Phytophthora* species. The horizontal black line within each box indicates the median value. K2P, pairwise genetic distances between each pair of the 34 core PCGs in the 11 *Phytophthora* mitogenomes based on the Kimura-2-parameter model; *Ka*, the number of nonsynonymous substitutions per nonsynonymous site; *Ks*, the number of synonymous substitutions per synonymous site.

To evaluate selective pressures, we calculated genetic distances and substitution rates for the core PCGs (Fig. 5). Among them, *rpl6* exhibited the highest non-synonymous substitution rate (Ka, mean = 0.0441), followed by *rps3* (mean = 0.0327), while *atp1* showed the lowest (mean = 0.0034). For synonymous substitutions, *rps4* had the highest Ks value (mean = 0.5719) and *nad7* followed (mean = 0.4340), whereas *rps13* was the lowest (mean = 0.0894). The Ka/Ks ratios for all genes fell well below 1, ranging from 0.0096 (*nad4L*) to 0.1737 (*rpl6*), confirming that purifying selection is the main driving force shaping *Phytophthora* mitogenomes. *rpl6* (0.1737), *rpl5* (0.1704), and *rps8* (0.1686) had the highest Ka/Ks ratios, indicating weaker purifying selection on these ribosomal genes (Suppl. material 1: table S13). Conversely, core respiratory genes like *nad4L* (0.0096), *atp1* (0.0185), and *cox1* (0.0172) showed exceptionally low ratios, reflecting strict functional conservation. Independent K2P genetic distance calculations confirmed these trends: *rpl6* showed the highest divergence (mean = 0.0792), followed by *rps2* (mean = 0.0726) and *nad11* (mean = 0.0721), while *nad3* was the most conserved (mean = 0.0221).

### Gene arrangement

**Figure 6.**
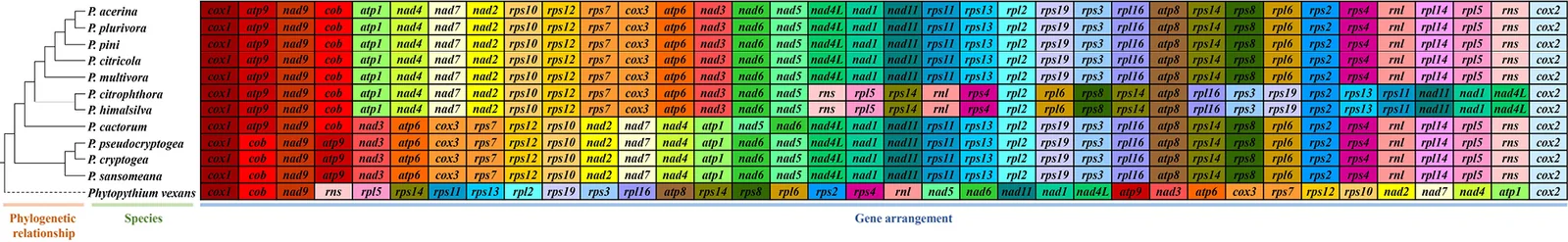
Mitochondrial gene arrangement analyses of the 11 *Phytophthora* species. The gene sequence begins with the cox1 gene and contains 34 core protein coding genes (PCGs) and two rRNA genes. Identical genes are represented by matching color blocks. Phylogenetic positions of the 11 species were established using the Bayesian inference (BI) method and Maximum-Likelihood (ML) method based on combined mitochondrial datasets.

In the present study, we analyzed the gene arrangements of 34 core PCGs and two rRNAs in the 11 *Phytophthora* mitogenomes. The results showed that all 11 *Phytophthora* mitochondrial genomes began with the *cox1* gene and ended with the *cox2* gene, with a complete and conserved gene cluster consistently maintained between these two genes (Fig. 6). However, based on differences in gene arrangement homology, the 11 species could be classified into three major arrangement types. Type I (conserved type) included five species: *P. acerina*, *P. plurivora*, *P. pini*, *P. citricola*, and *P. multivora*. These species exhibited completely identical gene orders, and this arrangement pattern was fully conserved within the Subclade 2c, reflecting an ancestral state inherited from their shared common ancestor. Type II (rearranged type) included *P. citrophthora* and *P. himalsilva*, both of which belong to Clade 2a. Compared with Type I, these two species exhibited a distinct inversion and rearrangement of the gene block located between *nad5* and *cox2*. The genes *rns*, *rpl5*, *rps14*, *rnl*, *rps4*, *rpl2*, and *rpl6*, which were originally located downstream, were collectively translocated to the region between *nad5* and *nad4L*. Although the order of the remaining genes was largely consistent with that of Type I, the overall arrangement pattern showed clear repositioning of several gene clusters. Type III (inverted type) included four species: *P. cactorum*, *P. pseudocryptogea*, *P. cryptogea*, and *P. sansomeana*. The gene arrangements of these species differed substantially from those of the previous two arrangement types and were primarily characterized by large-scale inversions. Specifically, the genomic region between *nad3* and *atp1* exhibited a complete inversion relative to Types I and II, whereas the arrangement of the remaining genes generally aligned with that of Type I. Within Type III, the gene arrangements of *P. pseudocryptogea*, *P. cryptogea*, and *P. sansomeana* were highly similar, whereas *P. cactorum* exhibited slight differences in the arrangement of several gene pairs, which aligns with their respective phylogenetic placements in Clade 8a and Clade 1a. The results showed that the positions of *cob* and *atp9* were exchanged, and the *nad5*–*nad6*

gene pair was inverted relative to the other three species. As the outgroup species, *Phytopythium vexans* exhibited a markedly different mitochondrial gene order. Notably, the gene block between *atp9* and *atp1* was rearranged and relocated upstream of the *cox2* gene. In addition, compared with the mitochondrial genomes of *Phytophthora* species, the genomic region between *atp8* and *rnl* was repositioned to the middle of the genome, and numerous ribosomal protein genes were concentrated in the front portion of the genome, resulting in low synteny with all examined *Phytophthora* species.

### Mitogenome rearrangements and multiple-genome collinearity

To further validate these gene arrangement patterns at the whole-genome level, multiple-genome collinearity analysis was performed, which identified five main locally collinear blocks (LCBs), labeled A–E (Suppl. material 2: fig. S2). Overall, the LCB genomic architecture strongly mirrored the three gene arrangement types defined above. Species in Subclade 2c (Type I) shared highly uniform LCB sequence compositions, lengths, and genomic positions, confirming exceptional structural stability with minimal rearrangements during their evolution. In contrast, species representing Types II and III (e.g., *P. cactorum*, *P. himalsilva*, *P. citrophthora*, *P. cryptogea*, and *P. pseudocryptogea*) exhibited marked deviations, characterized by rearrangements in LCB order and orientation, alongside localized fragmentation or expansions. Notably, Region C was significantly larger in *P. plurivora*, *P. pini*, *P. citricola*, *P. multivora*, *P. cryptogea*, and *P. sansomeana*, pointing to lineage-specific intergenic expansion and contraction events.

Relative to *Phytophthora*, the outgroup *Phytopythium vexans* displayed a completely altered LCB landscape with low overall synteny. Its *atp9*–*atp1* block was relocated upstream of *cox2*, the *atp8*–*rnl* region shifted to the middle of the genome, and numerous ribosomal protein genes clustered at the front portion, consistent with its deep phylogenetic divergence.

### Phylogenetic analysis, divergence times and structural dynamics

**Figure 7.**
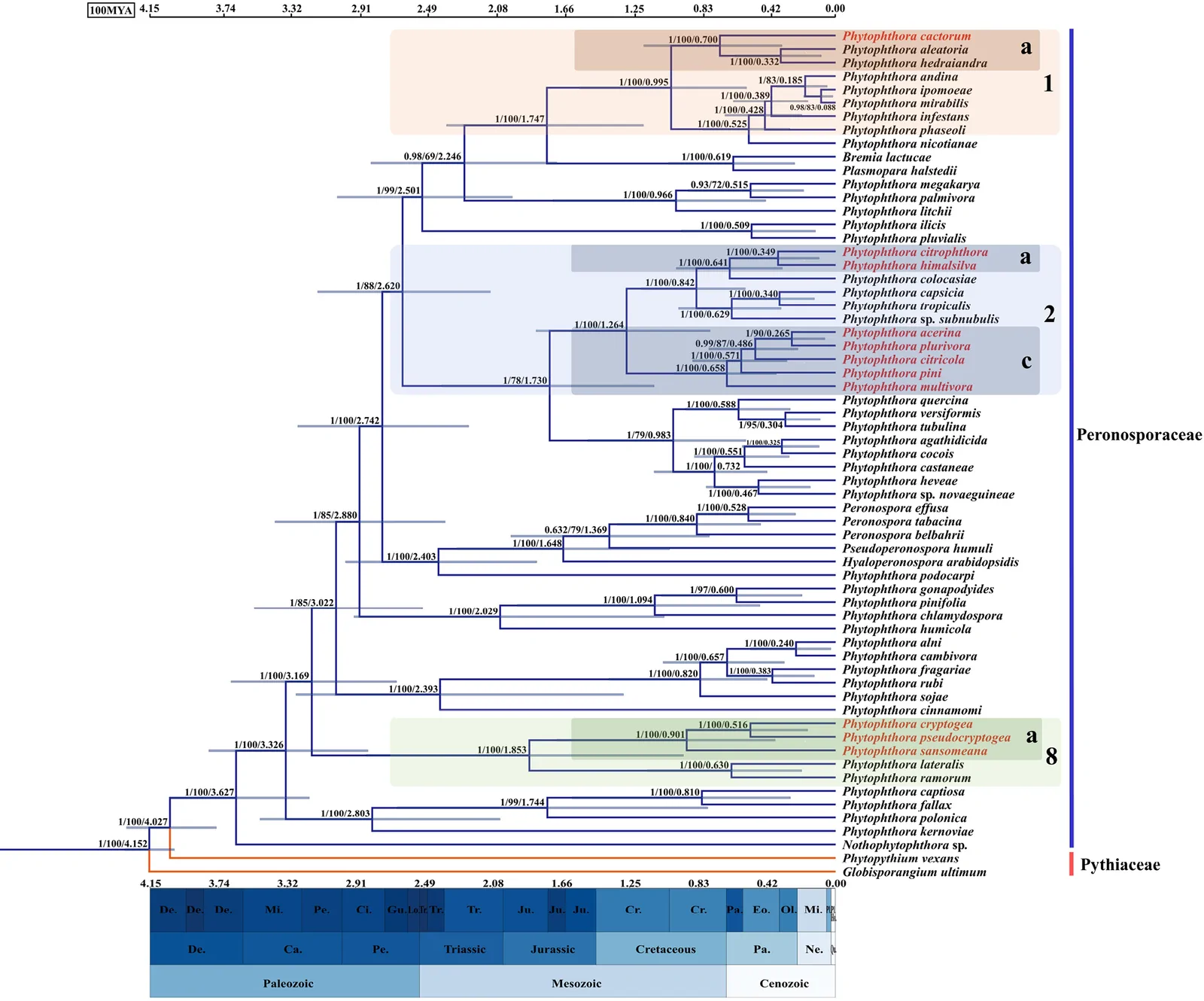
Time-calibrated phylogeny of 63 oomycete species inferred from a concatenated dataset of 34 mitochondrial protein-coding genes. The phylogenetic tree is rooted with *Globisporangium ultimum* and *Phytopythium vexans* as outgroups. Numbers at the internal nodes represent Bayesian posterior probabilities (PP), Maximum Likelihood bootstrap support values (BS, %), and estimated divergence times (in 100 MYA), respectively. Light blue horizontal bars indicate the 95% highest posterior density (HPD) intervals for node ages. The time scale at the top and bottom (in 100 million years ago, 100 MYA) is aligned with the geological time scale spanning from the Paleozoic (Devonian) to the Cenozoic, illustrating the deep evolutionary timeframe of lineage diversification. Taxa highlighted in bold red indicate the 11 target *Phytophthora* species analyzed in this study. (Abbreviations for geological eras and periods: De., Devonian; Ca., Carboniferous; Pe., Permian; Tr., Triassic; Ju., Jurassic; Cr., Cretaceous; Pa., Paleogene; Ne., Neogene; Eo., Eocene; Ol., Oligocene; Mi., Miocene).

**Figure 8.**
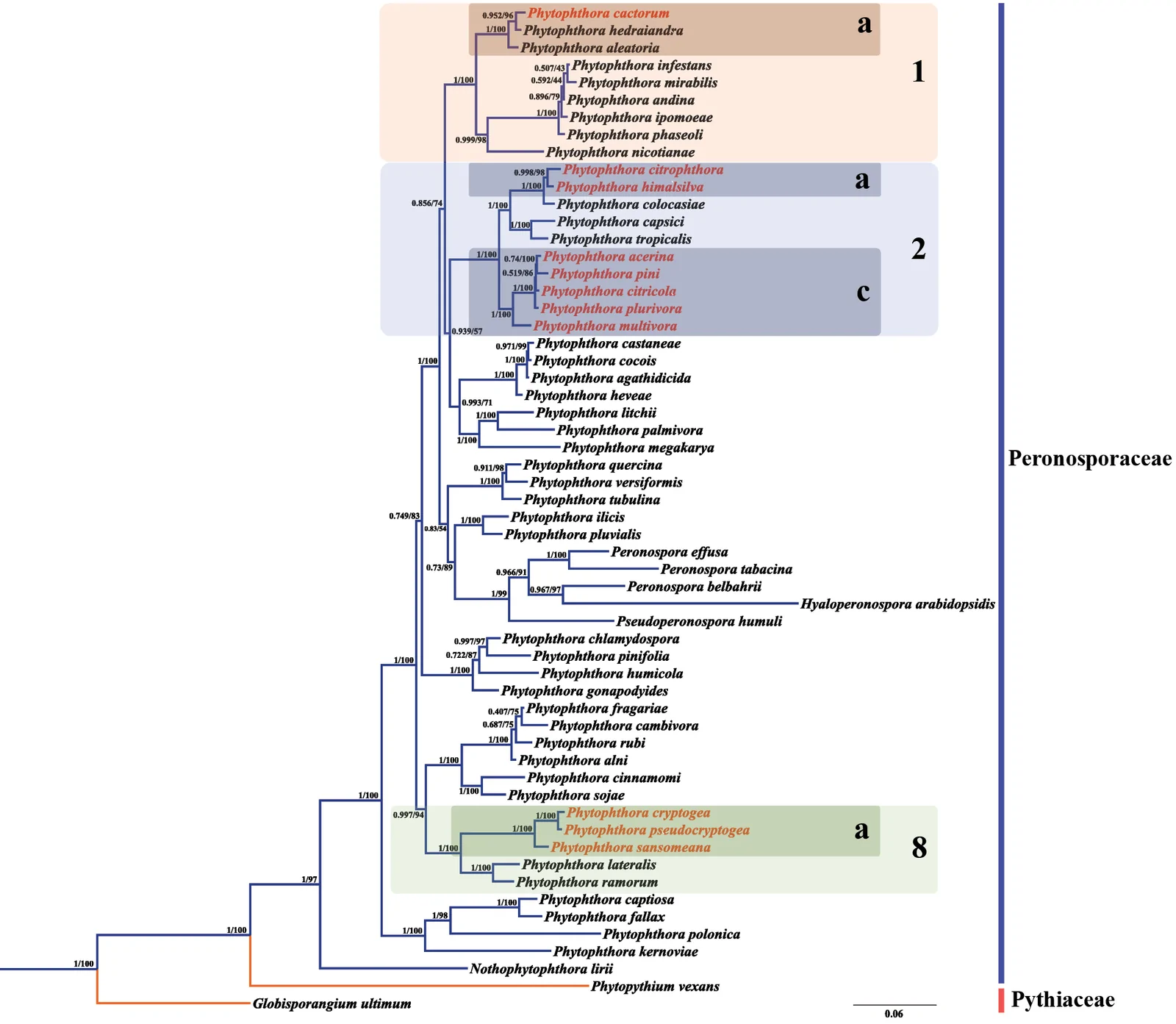
Phylogenetic tree of 58 oomycete species inferred from a concatenated dataset of five nuclear gene fragments. The topology was reconstructed using Maximum Likelihood (ML) and Bayesian Inference (BI) methods based on concatenated sequences of five genes. The phylogenetic tree is rooted with *Globisporangium ultimum* and *Phytopythium vexans* as outgroups. Numbers at the nodes represent Bayesian posterior probabilities (PP) and maximum likelihood bootstrap support values (BS), respectively. Taxa highlighted in bold red represent the target species focused on in this study.

Phylogenetic trees derived from the 34 core PCGs and multi-locus DNA markers yielded highly congruent major clades (Clades 1, 2, and 8) with robust statistical support (Figs 7, 8). However, minor topological discrepancies arose at terminal branches. In Clade 2, *P. citrophthora* and *P. himalsilva* formed a sister group (Subclade 2a) in both trees. Yet, within the *P. citricola* complex (subclade 2c), *P. acerina* clustered as a sister to *P. plurivora* in the mitochondrial tree, but placed closer to *P. pini* in the multi-locus tree. In Clade 8, *P. cryptogea* and *P. pseudocryptogea* consistently formed a stable sister-group with very short branch lengths, merging with *P. sansomeana* into a strongly supported monophyletic lineage. Notably, key nodes within Subclade 2c exhibited “strong conflicts” where both datasets supported mutually exclusive topologies with high confidence, highlighting the distinct evolutionary histories captured by maternally inherited mitogenomes versus biparentally inherited nuclear markers.

Molecular clock analysis using the 34 core PCGs estimated that the deep evolutionary split between *Phytophthora* and its pythiaceous relative *Globisporangium ultimum* occurred during the Devonian Period of the Paleozoic Era (~415 Ma) (Fig. 7). Major diversification events establishing the core *Phytophthora* crown clades (including Clades 1, 2, and 8) unfolded across the late Paleozoic to early Mesozoic (~250–300 Ma). Lineage diversification within individual clades (e.g., Subclades 1a, 2a, 2c, and 8a) progressed throughout the Jurassic and Cretaceous periods (~65–185 Ma). Specifically, Subclade 2c species exhibited more recent diversification events transitioning from the late Cretaceous into the Cenozoic, reflecting a relatively dynamic evolutionary radiation.

To trace structural history, we used the early-diverging *P. sansomeana* as an ancestral baseline reference (Suppl. material 2: fig. S3). This comparison showed that mitogenome size variation was primarily driven by the expansion and contraction of intergenic regions, while coding sequences remained highly static. Although PCG and ncRNA lengths fluctuated slightly across species relative to *P. sansomeana*, intergenic regions shifted dramatically.

Furthermore, divergence dating uncovered a cumulative temporal effect on mitogenome structures, where later-diverging lineages generally accumulated more intergenic sequences. For instance, the late-diverging *P. cryptogea* underwent extensive intergenic expansion, resulting in the largest mitogenome among the 11 species. Conversely, *P. citrophthora* and *P. acerina* retained shorter intergenic spaces and compact mitogenomes, indicating localized sequence contractions or fragment losses after split events.

## Discussion

### Size variation and compaction of *Phytophthora* mitogenomes

Mitogenome sizes among the 11 *Phytophthora* species ranged from 37.5 to 41.5 kb. This narrow (~10%) variation indicated moderate size divergence, which is relatively conservative within oomycetes and consistent with previously reported data for *Phytophthora* and related taxa (Winkworth et al. 2022). However, these genomes were substantially smaller than those of related *Pythiales* species, such as *Pythium insidiosum* (54,989 bp) and *Globisporangium ultimum* (59,689 bp) (Lévesque et al. 2010; Tangphatsornruang et al. 2016). This disparity aligned with findings that *Pythiales* and *Saprolegniales* had greater genomic complexity and more gene duplications than *Peronosporales* (O’Brien et al. 2014), reflecting the highly compact nature of *Phytophthora* mitogenomes (Cai and Scofield 2020). Notably, *P. cryptogea* showed an expanded mitogenome. Collinearity analysis suggested this expansion was driven by the enlargement of Region C within the locally collinear blocks (LCBs). Moreover, genome composition comparisons revealed that this size expansion stems from intergenic region enlargement or lineage-specific insertions rather than core gene amplification (Suppl. material 2: fig. S4). Thus, intra-genus mitogenome size variation in *Phytophthora* primarily reflects structural dynamics in non-coding regions rather than functional gene proliferation.

Additionally, all examined mitogenomes retained highly conserved circular structures and a stable gene complement (34 core PCGs, two rRNAs, and 25 tRNAs), consistent with previous *Phytophthora* studies (Paquin et al. 1997; Avila-Adame et al. 2006; Martin and Richardson 2007). This rigid structural conservation highlighted substantial functional constraints during evolution. Specifically, the consistent retention of oxidative phosphorylation genes (*cox*, *cob*, and *nad*) indicated highly conserved energy metabolism within the genus.

All mitogenomes exhibited an extreme AT bias (~22% GC content), a hallmark feature of oomycetes (Martin et al. 2007). While the precise underlying driver remains to be directly tested, this nucleotide bias is hypothesized to result from AT-biased mutational pressure during DNA replication or repair, or selective advantages related to replication efficiency and transcriptional regulation (Kenigsberg et al. 2016; Foster et al. 2018). Notably, similar strong AT biases (often with GC content <30%) are also widely observed in extant eubacterial groups, particularly obligate endosymbiotic or intracellular lineages such as *Rickettsiales* and *Mycoplasma* species, where they are primarily driven by severe genome reduction, loss of specific DNA repair pathways, and bioenergetic constraints (Moran et al. 2008; McCutcheon and Moran 2012). Given that mitochondria originated from an ancestral free-living eubacterium via endosymbiosis, it is plausible that the pervasive AT richness in *Phytophthora* mitogenomes likely reflects conserved mutational dynamics common to streamlined, endosymbiotic-like genomes. Compositional analysis showed that most *Phytophthora* species exhibited positive GC and AT skews, a pattern traditionally linked to strand-specific mutation or asymmetric replication. Notably, *P. himalsilva* and *P. citrophthora* exhibited a complete reversal to negative GC and AT skews. Although direct mechanisms were not experimentally verified in this study, this shift might be associated with potential alterations in replication origin location, replication orientation, or lineage-specific mutational pressures, though local rearrangements or altered selective constraints cannot be excluded. Similar skew patterns were obtained in other species (Massip et al. 2018). Therefore, while *Phytophthora* mitogenomes were universally AT-enriched, their base composition skews retained lineage-specific evolutionary plasticity.

Another prominent feature was the complete absence of introns across all 11 mitogenomes. Unlike fungal mitogenomes where introns were widespread and drove genome expansion, complete intron loss in *Phytophthora* points toward a history of extensive genome compaction. Although hypotheses suggest that this streamlined structure enhances replication efficiency or reduces energy expenditure during rapid growth and environmental adaptation (Martin et al. 2007; Yuan et al. 2017), it remains to be conclusively proven whether these selective pressures directly drove the compact structure of *Phytophthora* mitochondrial genomes. In summary, despite minor size variations among species, *Phytophthora* mitogenomes consistently exhibited high structural conservation, extreme AT enrichment, and complete intron loss, patterns that are consistent with highly compact architectures maintained under rigid evolutionary constraints.

### Codon usage patterns and purifying selection on PCGs

This study demonstrated that mitochondrial PCGs across the 11 *Phytophthora* species exclusively used ATG as the start codon and exhibited a profound A/T bias in stop and synonymous codon usage. This extreme preference aligned with the low genomic GC content (22%), indicating that codon usage was primarily driven by mutational pressure rather than translational selection (Iyyappan et al. 2025; Amenu et al. 2026). In an AT-enriched genomic context, mutations preferentially accumulated A/T bases, enriching codons ending in A or T—a phenomenon widespread in oomycete mitogenomes (Lin et al. 2025). Notably, stop codon usage varied among genes and lineages; *nad11* consistently used TGA across all species, whereas TAG stop codons were exclusively detected within Subclade 2c. This lineage-specific pattern suggests that stop codon variation reflects both mutational preferences and phylogenetic divergence, highlighting its utility as a phylogenetic marker (Xu et al. 2012; Qi et al. 2026).

Ka/Ks analysis revealed that Ka/Ks ratios for all 34 PCGs were significantly below 1 (0.0096–0.1737), demonstrating that purifying selection was the dominant evolutionary force shaping *Phytophthora* mitogenomes (Cai and Scofield 2020). This constraint stems from the critical role of mitochondria in energy metabolism; mitochondrially encoded proteins are indispensable for oxidative phosphorylation (OXPHOS), electron transport, and ATP synthesis (Hock et al. 2020; Fernández‐Vizarra and Zeviani 2020). Consequently, these genes face stringent functional constraints to maintain metabolic stability. Nevertheless, different gene categories exhibited distinct functional differentiation. Core respiratory chain genes, such as *nad4L* (Ka/Ks = 0.0096), *cox1* (0.0172), and *atp1* (0.0185), showed extremely low Ka/Ks values. Because these proteins are directly involved in electron transport and proton gradient formation, even minor amino acid substitutions could impair energy metabolism. In contrast, ribosomal protein genes (*rpl6*, *rpl5*, and *rps8*) exhibited higher Ka/Ks values (all less than 1), indicating weaker purifying selection. This pattern likely reflects the greater structural redundancy or functional flexibility of ribosomal proteins, which typically occupy peripheral regions of protein complexes where structural variations are better tolerated (Lindahl 2024; Helena-Bueno et al. 2025). Nevertheless, a methodological limitation of our selection analysis lies in evaluating evolutionary constraints via gene-wide pairwise Ka/Ks ratios. Although average Ka/Ks values below 1 reflect strong purifying selection, whole-gene averaging can dilute localized adaptive signals acting on specific codon sites or motifs. Therefore, these pairwise estimates should be interpreted as baseline evolutionary trends rather than evidence for an absolute absence of positive selection. Future studies employing site-specific selection models and denser population sampling are needed to identify specific target codons driving environmental adaptation.

K2P genetic distance analysis yielded results highly congruent with the Ka/Ks patterns, confirming the reliability of these evolutionary trends. Results showed that *rpl6* exhibited both the highest Ka/Ks value and the largest genetic distance, indicating accelerated sequence divergence. Conversely, *nad3* and *cox1* showed minimal genetic distances, reinforcing their highly conserved nature. This consistency shows that selective pressure and sequence divergence jointly reflect gene-specific evolutionary rates. Furthermore, evolutionary constraints correlated with nucleotide composition; genes with higher GC content (e.g., *cox1* and *atp9*) tended to exhibit lower Ka/Ks values, potentially due to the structural stability and functional importance of GC-rich regions (Cai and Scofield 2020). Additionally, most genes displayed a negative AT skew and a positive GC skew, reflecting replication and mutation asymmetries that shape nucleotide composition, codon usage preferences, and overall evolutionary rates (Eberhard and Wright 2015; Ye et al. 2022).

In summary, 11 *Phytophthora* mitochondrial PCGs evolved under a regime of general conservation accompanied by localized variation. Stringent purifying selection maintained core gene stability, whereas variation in specific ribosomal genes and stop codons provided phylogenetically informative signals. This differential evolutionary pattern reflects hierarchical functional constraints and offers valuable tools for phylogenetic reconstruction.

### Three major types of mitogenome rearrangements and non-linear evolution

Based on the alignment of 34 core PCGs and rRNA genes, this study systematically classified the mitogenomes of 11 *Phytophthora* species into three major arrangement types (conservative, rearranged, and inverted), providing novel insights into the clade-specific structural dynamics of this genus. Specifically, Subclade 2c (i.e., *P. pini* and *P. plurivora*) exhibited an extremely high degree of structural conservation (Fig. 6). Both gene order and locally collinear blocks (LCBs) were highly congruent within this clade, with virtually no rearrangement or translocation events detected. While the precise underlying mechanisms remain to be directly tested, this remarkable stability may reflect lower recombination frequencies, weaker structural mutational pressure (Dong et al. 2019; Cai and Scofield 2020), or stringent functional constraints that preserve gene order once established (Yuan et al. 2017; Lin et al. 2025). Furthermore, it is plausible that stable gene organization may be loosely constrained by replication origins or transcriptional regulatory structures, potentially limiting large-scale structural changes. In contrast, our results revealed that mitogenomic evolution in other clades was far from static. Subclade 2a (i.e., *P. citrophthora* and *P. himalsilva*) displayed typical rearrangement-type characteristics, primarily manifested as the large-scale translocation of specific gene clusters, including rRNA and ribosomal protein genes. This block-like translocation pattern demonstrates that structural variations in these lineages were likely driven by macromutations or macro-rearrangements rather than random, single-gene movements, pointing to a previously underestimated mode of structural evolution.

Furthermore, inversion-type lineages (i.e., *P. cryptogea*, *P. pseudocryptogea*, *P. sansomeana*, and *P. cactorum*) exhibited even more dramatic variations, highlighted by a complete inversion of the *nad3*–*atp1* region. Although similar rearrangement patterns have been documented in other *Phytophthora* mitogenomes (Martin et al. 2007; Cai and Scofield 2020), our fine-scale comparison further identifies species-specific gene position exchanges (e.g., *cob* and *atp9*) within this inverted type, emphasizing lineage-specific structural divergence. In line with previous evolutionary models, such large-scale inversions are often hypothesized to be associated with replication-related recombination and may be mediated by repetitive sequences. Coupled with the LCB fragmentation observed here, these variations might tentatively stem from repeat-mediated recombination or mobile genetic elements, although we emphasize that direct functional evidence for these mechanisms in oomycete mitochondria remains to be empirically established (Zhong et al. 2022; Kong et al. 2025). Together, these findings demonstrate that *Phytophthora* mitogenomes evolve through non-linear, recombination-driven structural reorganizations across different phylogenetic lineages.

### Collinearity conservation and lineage-specific genome diversification

Mauve collinearity analysis further expanded our understanding of these structural dynamics, demonstrating that mitogenome evolution in *Phytophthora* does not follow a simple linear path. *Phytophthora cactorum* (Subclade 1a) displayed a unique “mosaic-like” arrangement, containing Region B (shared with Subclade 2c) while retaining Region G (found in Subclades 2a and 8a). This hybrid architecture suggests that the *P. cactorum* mitogenome either retained ancestral structural features common to multiple lineages or underwent independent, parallel rearrangements during evolution. Notably, comparative evaluation with the published mitogenome of *Phytophthora infestans* from Subclade 1c (Martin et al. 2007) revealed an extremely high degree of gene order conservation with *P. cactorum*. The two species share an almost identical structural backbone across the entire mitogenome, differing only by a localized inversion within the *atp1*–*nad3* gene cluster. This remarkably conserved architecture within Clade 1 suggests that major structural block reorganizations predominantly occur between more distantly related clades, whereas intra-clade mitogenome evolution is constrained to minor, localized recombination events. On a macro level, this complex pattern implies that mitogenome structural evolution involves intricate genomic fragment recombination and rearrangement rather than a straight phylogenetic trajectory (Mandal et al. 2022; Lin et al. 2025). Notably, although *P. cryptogea* (Subclade 8a) phylogenetically clustered outside Subclade 2c, its mitogenome structure was more similar to that of Subclade 2c species than to other non-2c species examined here (i.e., *P. cactorum*, *P. citrophthora*, *P. himalsilva*, *P. pseudocryptogea*, and *P. sansomeana*). This discrepancy indicates that mitogenome structural evolution does not always align with sequence-based phylogenetic relationships. Such incongruence may stem from the independent retention of a conserved ancestral structural state across divergent lineages (Popova et al. 2016; DeSalle and Tessler 2025). Alternatively, convergent structural evolution cannot be ruled out, whereby distinct lineages independently develop similar gene arrangements under equivalent selective pressures or via repeat-mediated recombination (Maier et al. 2013; Montaña-Lozano et al. 2022).

In cross-species comparisons, the frequency of mitogenome rearrangements across the 11 *Phytophthora* species appeared lower than in related genera like *Globisporangium* and *Phytopythium*, indicating higher intra-genus structural stability (Lévesque et al. 2010). *Ph. vexans*, used as the outgroup, exhibited markedly different gene arrangements, including large-scale block translocations and ribosomal protein gene redistribution, resulting in low collinearity with *Phytophthora* species. This divergence suggests that mitogenome structure evolves rapidly at higher taxonomic levels but remains relatively stable within *Phytophthora*, carrying substantial phylogenetic and evolutionary significance.

### Topological conflicts between mitogenomic and multi-locus trees

This study separately constructed phylogenetic trees using mitochondrial core PCGs and multi-locus gene fragments (ITS, *ypt1, tigA, cox1* and *rps10*). Combined with mitogenome structural analysis, a clear correlation was observed between phylogenetic relationships and genomic structural variations (Kroon et al. 2004; Martin et al. 2014). Subclade 2c formed a stable monophyletic group in both the mitochondrial and multi-locus trees and exhibited highly consistent gene arrangements, indicating that this clade has remained structurally conservative during mitogenome evolution (Martin and Tooley 2003). In contrast, *P. cactorum* and *P. cryptogea* exhibited more complex rearrangement patterns and relatively independent phylogenetic positions, suggesting that mitogenome structural remodeling occurs concurrently with phylogenetic divergence (Cai and Scofield 2020; Lin et al. 2025). Significantly, this study identified several highly supported topological conflicts between the mitochondrial and multi-locus trees. At several nodes within Subclade 2c, both trees supported mutually exclusive topologies with high confidence, ruling out false conflicts caused by random error or insufficient phylogenetic signals. This incongruence typically indicates that different genomic compartments recorded distinct evolutionary histories. Because mitogenomes are typically uniparentally inherited whereas multi-locus markers reflect biparental inheritance, inter-lineage hybridization and introgression may represent plausible drivers of these topological conflicts (Turissini et al. 2017; Secci‐Petretto et al. 2022; Quattrini et al. 2023).

The mito-nuclear discordance observed among these non-hybrid *Phytophthora* lineages is most plausibly explained by incomplete lineage sorting (ILS) during rapid divergence. Our molecular clock analysis indicated that species within Subclade 2c diverged relatively recently, with multiple divergence events concentrated during the Miocene, suggesting rapid radiation. During rapid divergence, ancestral polymorphisms may not completely sort prior to speciation, causing different genomic compartments to retain conflicting ancestral genetic signals (Linnen and Farrell 2007; Van Poucke et al. 2021; Quattrini et al. 2023; Jung et al. 2024). Consequently, mitochondrial and nuclear genomes preserved discordant phylogenetic histories, yielding highly supported yet conflicting topologies. However, because our current dataset lacks population-level coalescent-based modeling or genome-wide introgression analyses, definitively distinguishing among subtle ancestral evolutionary processes remains challenging. Thus, these explanations warrant further verification via genome-wide data. Therefore, we inferred that structural variations in the mitogenome do not accumulate randomly; instead, they represent evolutionary features that emerge and become stably fixed during lineage divergence. Consequently, gene arrangements, repeat distributions, and syntenic variations all carry crucial phylogenetic signals, serving as valuable complements to traditional sequence-based phylogenetics.

### Mitogenome structural remodeling and lineage-specific evolutionary rates

Integrating our structural analysis into a temporal framework reveals that *Phytophthora* mitogenome evolution is not a uniform, linear progression, but rather a dynamic process marked by lineage-specific rate heterogeneity. Compared to earlier-diverging lineages, the Subclade 2c lineage (comprising *P. plurivora*, *P. pini*, *P. citricola*, and *P. multivora*) diverged more recently and exhibits remarkable structural conservation, marked by minimal intergenic variation and strict gene synteny. From an adaptive standpoint, the preservation of conserved syntenic blocks confers a strong selective advantage by maintaining essential polycistronic transcriptional units and the co-regulation of core oxidative phosphorylation complexes, thereby preventing deleterious disruptions to mitochondrial energy metabolism. Temporally, this conservation also reflects time constraints, where insufficient time has elapsed for substantial repeat-mediated recombination or structural remodeling to accumulate and fix in recently diverged lineages.

Conversely, species in earlier-diverging clades display pronounced structural remodeling, including intergenic expansions, local rearrangements, and shifted collinear block orders. These structural shifts are initially seeded as chance evolutionary events driven by repeat-mediated non-homologous recombination and genetic drift operating over deep evolutionary timescales. However, rather than remaining purely neutral noise, such genomic remodeling—particularly intergenic expansions—may offer key regulatory plasticity by hosting novel promoter elements or functional non-coding RNAs, ultimately providing an adaptive advantage for coping with diverse host range and ecological niches. Overall, structural remodeling in *Phytophthora* mitogenomes reflects a dynamic balance between strong purifying selection on functional coding cores and adaptive flexibility across non-coding regions in divergent lineages (Lee et al. 2019; Fonseca et al. 2020; Najer et al. 2023).

Therefore, integrating these structural insights with our molecular clock analysis, we conclude that *Phytophthora* mitogenome structural evolution is neither a purely passive byproduct of time nor a solely selectionist process, but rather a dynamic continuum driven by the interplay between neutral genomic mechanics and evolutionary selection. While strong purifying selection maintains essential respiratory architecture (as observed in Subclade 2c), repeat-mediated recombination generates structural novelty over deep time, which is subsequently filtered by natural selection to facilitate regulatory adaptation. Consequently, the expansion and contraction of intergenic regions, alterations in collinearity blocks, and genomic rearrangements collectively constitute the primary mechanisms shaping *Phytophthora* mitogenomic evolution.

### Reevaluating oomycete evolutionary timelines: Biogeographical vs. Fossil calibrations

Accurately contextualizing these mitogenomic dynamics depends heavily on the choice of molecular clock calibration strategies. Divergence time estimations in *Oomycota* have historically varied depending on whether fossil or biogeographical constraints were applied. In our initial analysis using microfossil-based calibrations (Matari and Blair 2014), the divergence time between *Phytophthora* and other pythiaceous/peronosporaceous genera was estimated to be unrealistically young (~23.4–26.7 Mya; Suppl. material 2: fig. S5). As highlighted by critiques and studies (Krings et al. 2011; Jung et al. 2024), early microfossils lack diagnostic oomycete characters and could equally represent ancient fungal lineages, thereby severely underestimating divergence times. This Cenozoic timeline conflicts directly with modern biogeographical data showing that major *Phytophthora* clades (e.g., Clades 2c, 2e, and 2f) diverged prior to the breakup of Pangea into Gondwana and Laurasia (~200 Mya).

By re-calibrating our molecular clock using well-justified fossil and biogeographical constraints (Jung et al. 2022, 2024), our updated timeline places the origin of *Peronosporaceae* in the Devonian (~415 Ma) and the primary radiation of major *Phytophthora* lineages firmly across the late Paleozoic and Mesozoic Eras (~250–300 Ma) (Fig. 7). This revised ancient timetable provides a far more realistic chronological framework for the accumulation of mitogenomic structural variations across deep evolutionary times. Furthermore, our field recoveries of endemic Clade 2 species (*P. acerina*, *P. citricola*, *P. citrophthora*, *P. himalsilva*, and *P. plurivora*) in undisturbed habitats of the Qinling Mountains strongly corroborate East Asia as a primary center of origin and diversification for Clade 2 (Jung et al. 2024). Conversely, the co-occurrence of introduced pathogens such as *P. cactorum* and *P. multivora* points to historic anthropogenic movement, emphasizing the vital role of biogeographically informed evolutionary models in forest health management and global biosecurity.

## Conclusions

By integrating comparative mitogenomics, biogeographical molecular clock calibrations, and field recoveries, this study provides a comprehensive evolutionary synthesis of mitogenome architecture and lineage diversification across 11 representative *Phytophthora* species. Rather than reflecting simple passive sequence expansion, our findings indicate that organellar genome structural remodeling in *Phytophthora* is best explained by an interplay between functional purifying selection and neutral genomic mechanics. While core metabolic gene synteny is strictly conserved under strong selective constraints to preserve respiratory chain efficiency—as exemplified by Subclade 2c—intergenic region dynamics and localized rearrangements are continuously shaped by repeat-mediated recombination and genetic drift over deep evolutionary timescales.

Contextualizing these structural dynamics within ancient plate-tectonic calibrations establishes that major *Phytophthora* clades diverged during the late Paleozoic to early Mesozoic (~250–300 Ma), with key intra-clade diversification events (i.e., Clades 1, 2, and 8) unfolding throughout the Jurassic and Cretaceous periods, providing a realistic ancient timescale for mitogenomic structural divergence. Crucially, our field recoveries in the Qinling Mountains bridge theoretical phylogenomics with applied forest pathology by validating East Asia as the natural evolutionary origin for Clade 2 species, while simultaneously pinpointing human-mediated introductions of exotic pathogens. Ultimately, this work elevates oomycete mitogenomics from basic structural description to an integrative evolutionary framework, offering crucial foundations for tracing pathogen origins, understanding organellar dynamics, and safeguarding international plant biosecurity.

## Additional information

### Artificial Intelligence (AI) use

The authors accept full responsibility for the content of the manuscript, including the disclosure of any use of AI.

No AI tools were used in the preparation of this manuscript.

### Adherence to national and international regulations

All the fungal strains used in this study have been legally obtained, respecting the Convention on Biological Diversity (Rio Convention).

### Author ORCIDs

A. Hieno https://orcid.org/0000-0001-5019-2336

K. Kageyama https://orcid.org/0000-0003-2255-4920

M. Li https://orcid.org/0000-0001-8078-333X

### Data availability

The complete mitochondrial genome of Phytophthora species has been deposited in the GenBank database, and the accession numbers, along with all data generated or analyzed during this study are included in this article and its Suppl. materials 1.

## Supplementary materials

Supplementary material 1 Additional tables Data type xlsx Explanation note **table S1**. Geographic sampling locations, host tree species, and isolated *Phytophthora* strains. **table S2**. Accession numbers of the multigenic phylogeny genes. **table S3**. Read-mapping statistics, sequencing depth, and assembly validation metrics for the 11 *Phytophthora mitogenomesa*. **table S4**. Accession numbers of the mitochondrial genome. **table S5**. Comparison of mitogenomes among 11 species from *Phytophthora*. **table S6**. Annotation and characterization of the eleven *Phytophthora* mitogenomes. **table S7**. Distribution of variable sites in tRNA genes of eleven Phytophthora mitogenomes. **table S8**. Codon usage analysis of eleven *Phytophthora* mitogenomes. **table S9**. SSR detected in the mitogenomes of eleven *Phytophthora* species using MISA. **table S10**. Tandem repeats detected in the mitogenomes of eleven *Phytophthora* species. **table S11**. Local BLAST analysis of the eleven *Phytophthora* species mitogenomes against themselves. **table S12**. Core protein coding gene information of the eleven *Phytophthora* species. **table S13**. Tandem repeats detected in the mitogenomes of eleven *Phytophthora* species using the Tandem Repeats Finder. https://binary.pensoft.net/file/1750294This dataset is made available under the Open Database License (http://opendatacommons.org/licenses/odbl/1.0/). The Open Database License (ODbL) is a license agreement intended to allow users to freely share, modify, and use this Dataset while maintaining this same freedom for others, provided that the original source and author(s) are credited. Yijia Sun, Xiaowei Chen, Wenxin Wang, Yaling, Li, Ayaka Hieno, Koji Kageyama, Mingzhu Li

Supplementary material 2 Additional figures Data type pdf Explanation note **fig. S1**. Maximum Likelihood (ML) and Bayesian Inference (BI) phylogenetic tree of 101 oomycete species inferred from a concatenated dataset of five gene fragments (ITS-rDNA, *cox1*, *ypt1*, *rps10*, and *tigA*). **fig. S2**. Collinearity analysis of eleven *Phytophthora* mitogenomes as generated with Mauve 2.4.0. **fig. S3**. The proportion of different genetic compositions and their contribution to mitogenome contraction (above) and expansion (below) in eleven *Phytophthora* mitogenomes. **fig. S4**. Differences in genetic compositions and their contributions to mitogenome size variation among eleven *Phytophthora* species relative to the basal taxon *Phytophthora sansomeana*. **fig. S5**. Time-calibrated mitochondrial phylogeny based on early fossil calibrations. https://binary.pensoft.net/file/1750295This dataset is made available under the Open Database License (http://opendatacommons.org/licenses/odbl/1.0/). The Open Database License (ODbL) is a license agreement intended to allow users to freely share, modify, and use this Dataset while maintaining this same freedom for others, provided that the original source and author(s) are credited. Yijia Sun, Xiaowei Chen, Wenxin Wang, Yaling, Li, Ayaka Hieno, Koji Kageyama, Mingzhu Li
