# Supplementary material for: Mitochondrial genomic variation, evolutionary dynamics, and phylogeny of forest-associated Phytophthora species in the Qinling Mountains of China
Source: IMA Fungus. 2026 Aug 20;17:e203360. doi: 10.3897/imafungus.17.203360 (PMC13522877; doi:10.3897/imafungus.17.203360)
Supplement: Supplementary file 1 [file imafungus-17-e203360-s002.pdf]

Suppl. material 2

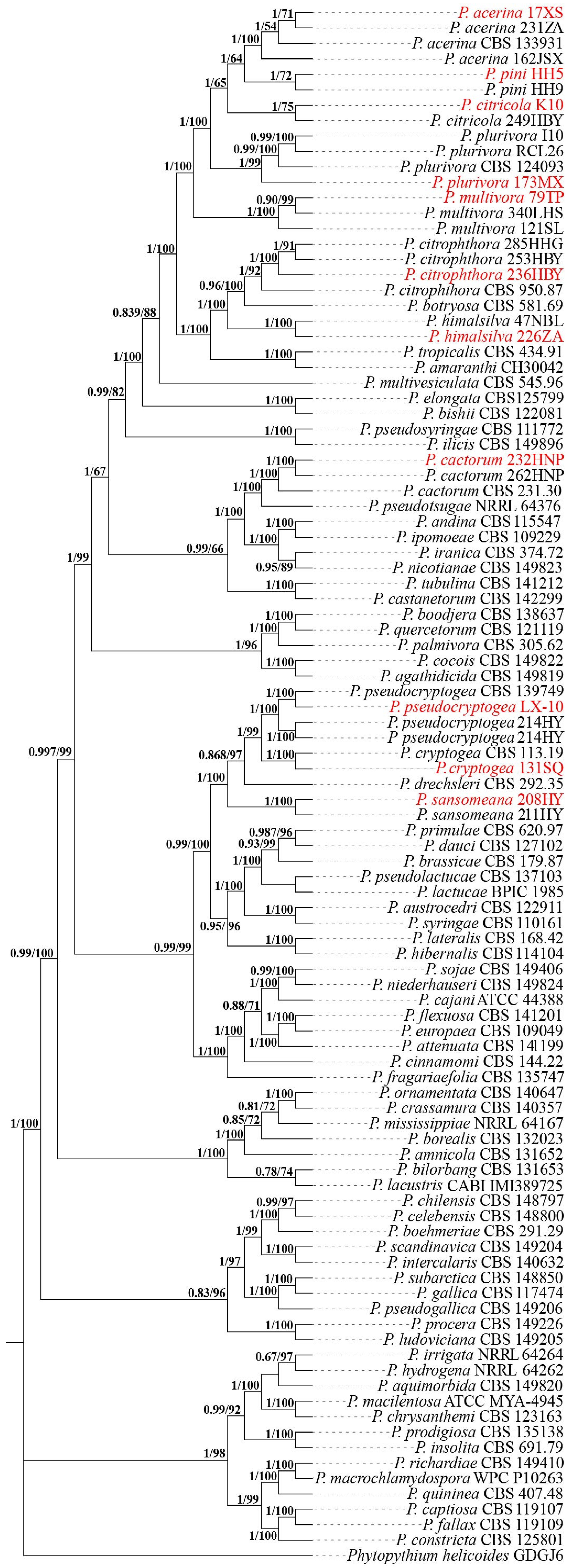

figure S1. Maximum Likelihood (ML) and Bayesian Inference (BI) phylogenetic tree of 101 oomycete species inferred from a concatenated dataset of five gene fragments (ITS-rDNA, *cox1*, *ypt1*, *rps10*, and *tigA*). The tree is rooted with *Phytopythium helicoides GDGJ6* as the outgroup. Numbers at nodes indicate Bayesian posterior probabilities (PP) and Maximum Likelihood bootstrap support values (BS), respectively. Target strains focused on in this study are highlighted in red.

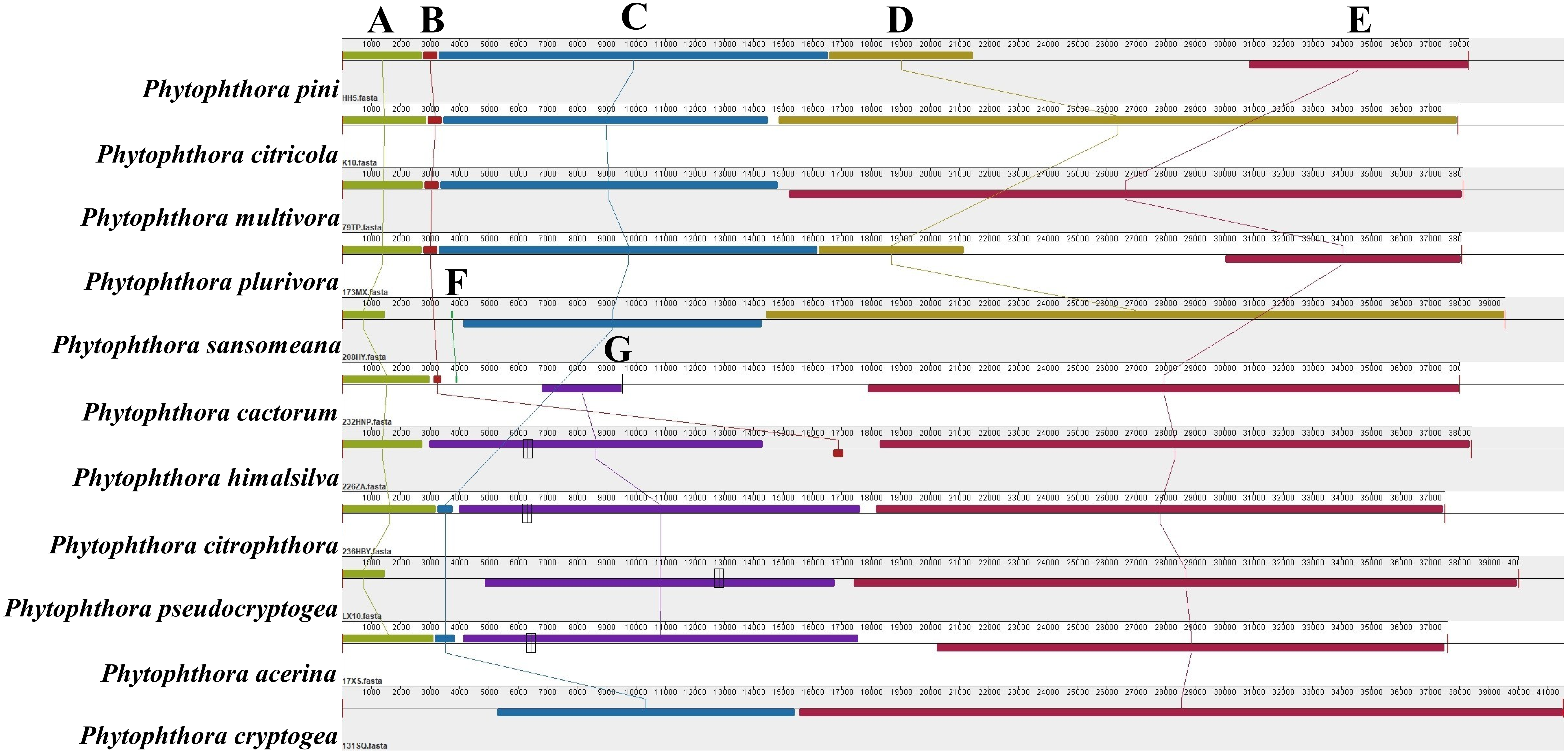

figure S2. Collinearity analysis of eleven *Phytophthora* mitogenomes as generated with Mauve 2.4.0. Homologous regions between different species were represented by the same color blocks and connected by the same color lines.

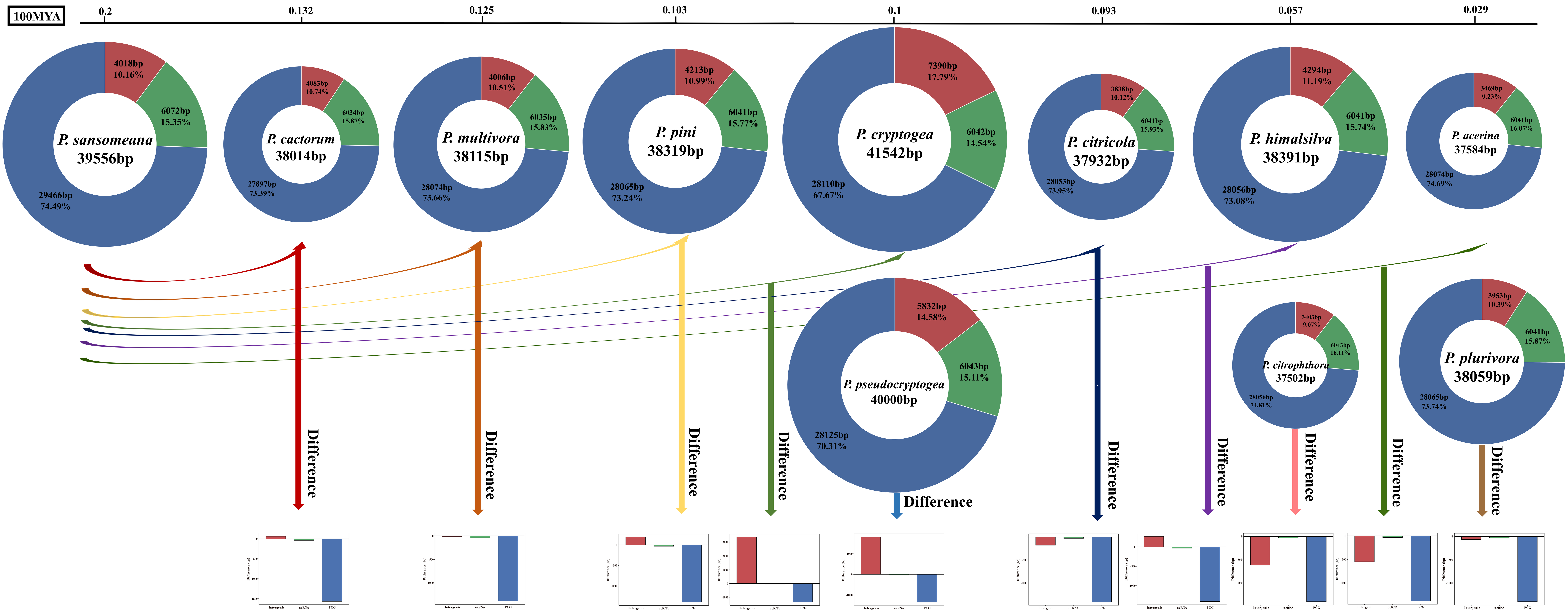

figure S3. Differences in genetic compositions and their contributions to mitogenome size variation among eleven *Phytophthora* species relative to the basal taxon *Phytophthora sansomeana*.

A

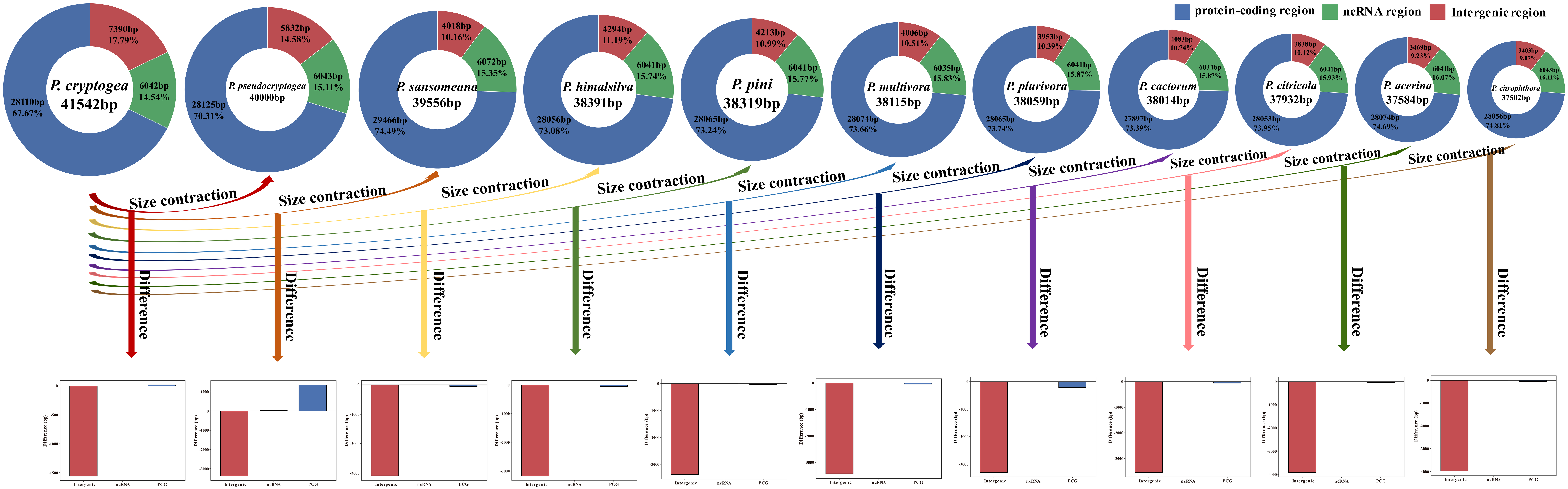

protein-coding region
ncRNA region
Intergenic region
P. cryptogea
41542bp
7390bp 17.79%
6042bp 14.54%
28110bp 67.67%
P. pseudocryptogea
40000bp
5832bp 14.58%
6043bp 15.11%
28125bp 70.31%
P. sansomeana
39556bp
4018bp 10.16%
6072bp 15.35%
29466bp 74.49%
P. himalsilva
38391bp
4294bp 11.19%
6041bp 15.74%
28056bp 73.08%
P. pini
38319bp
4213bp 10.99%
6041bp 15.77%
28065bp 73.24%
P. multivora
38115bp
4006bp 10.51%
6035bp 15.83%
28074bp 73.66%
P. plurivora
38059bp
3953bp 10.39%
6041bp 15.87%
28065bp 73.74%
P. cactorum
38014bp
4083bp 10.74%
6034bp 15.87%
27897bp 73.39%
P. citricola
37932bp
3838bp 10.12%
6041bp 15.93%
28053bp 73.95%
P. acerina
37584bp
3469bp 9.23%
6041bp 16.07%
28074bp 74.69%
P. citrophthora
37502bp
3403bp 9.07%
6043bp 16.11%
28056bp 74.81%
Size contraction
Difference
Difference (bp)
Intergenic
ncRNA
PCG

B

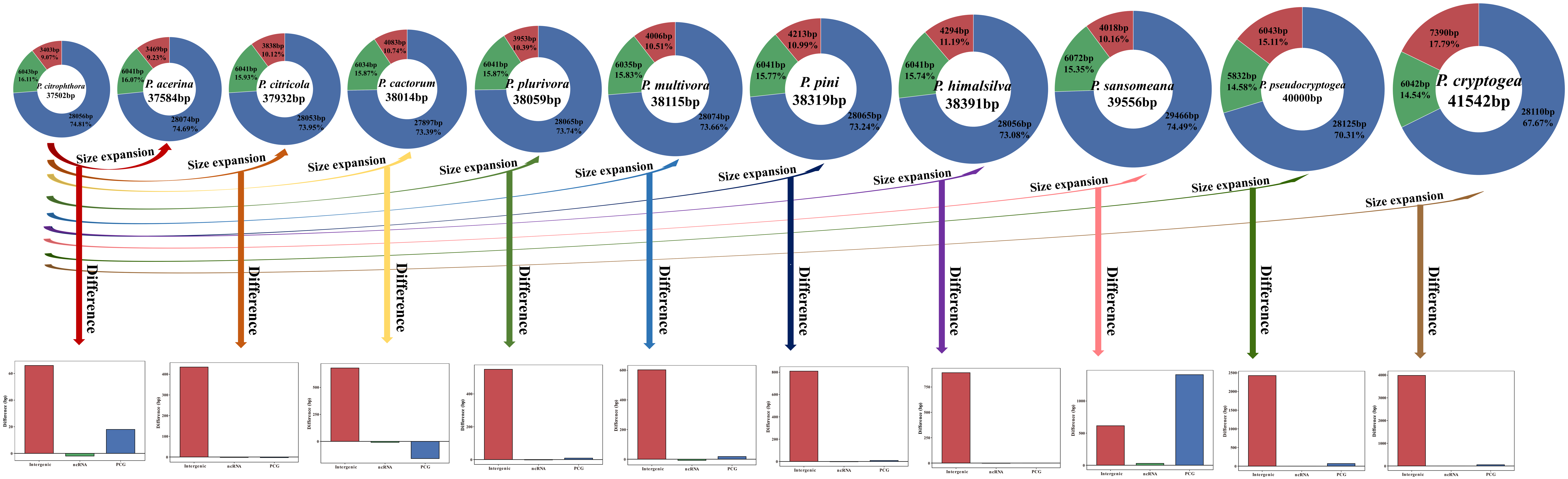

figure S4. The proportion of different genetic compositions and their contribution to mitogenome contraction (above) and expansion (below) in eleven *Phytophthora* mitogenomes.

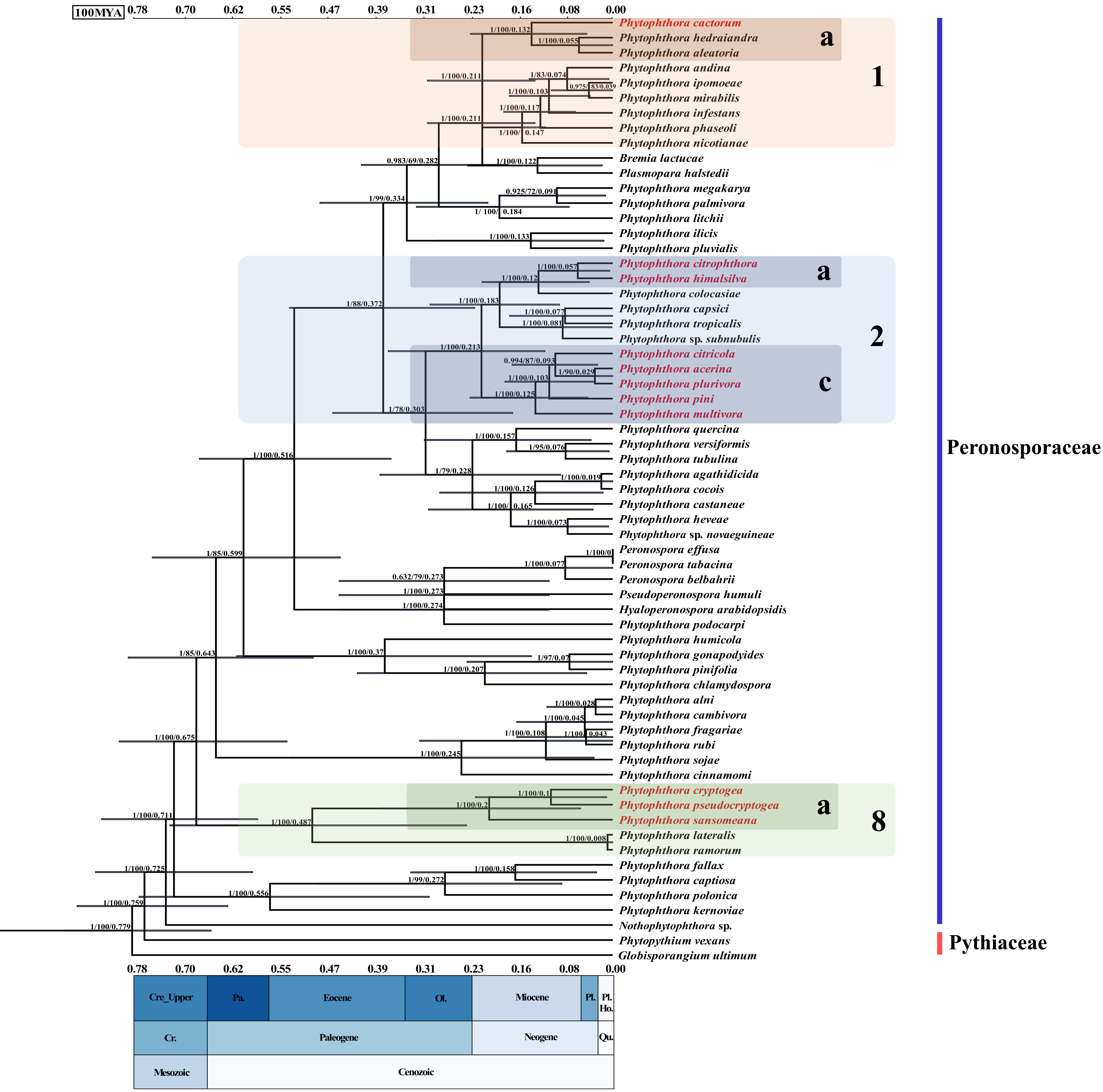

figure. S5 Time-calibrated mitochondrial phylogeny based on early fossil calibrations. Divergence times of 63 Oomycete species estimated using microfossil constraints from Matari and Blair (2014). Nodes show Bayesian posterior probabilities (PP), Maximum Likelihood bootstrap support (BS), and estimated mean divergence times (Ma), with grey bars indicating 95% HPD intervals. The scale at the bottom represents geological time in millions of years ago (Ma). Note the substantial underestimation of divergence dates across all major oomycete clades compared to biogeographical calibrations (Fig. 7). Target species focused on in this study are indicated in bold red text.
